# HOXA repression is mediated by nucleoporin Nup93 assisted by its interactors Nup188 and Nup205

**DOI:** 10.1186/s13072-016-0106-0

**Published:** 2016-12-03

**Authors:** Ajay S. Labade, Krishanpal Karmodiya, Kundan Sengupta

**Affiliations:** Biology, Indian Institute of Science Education and Research (IISER), Dr. Homi Bhabha Road, Pashan, Pune, Maharashtra 411008 India

**Keywords:** HOXA, Nuclear pore complex (NPC), Nup93, Nup188, Nup205, Nucleoporins (Nups), Chromatin, ChIP

## Abstract

**Background:**

The nuclear pore complex (NPC) mediates nuclear transport of RNA and proteins into and out of the nucleus. Certain nucleoporins have additional functions in chromatin organization and transcription regulation. Nup93 is a scaffold nucleoporin at the nuclear pore complex which is associated with human chromosomes 5, 7 and 16 and with the promoters of the HOXA gene as revealed by ChIP-on-chip studies using tiling microarrays for these chromosomes. However, the functional consequences of the association of Nup93 with HOXA is unknown.

**Results:**

Here, we examined the association of Nup93 with the HOXA gene cluster and its consequences on HOXA gene expression in diploid colorectal cancer cells (DLD1). Nup93 showed a specific enrichment ~1 Kb upstream of the transcription start site of each of the HOXA1, HOXA3 and HOXA5 promoters, respectively. Furthermore, the association of Nup93 with HOXA was assisted by its interacting partners Nup188 and Nup205. The depletion of the Nup93 sub-complex significantly upregulated HOXA gene expression levels. However, expression levels of a control gene locus (GLCCI1) on human chromosome 7 were unaffected. Three-dimensional fluorescence in situ hybridization (3D-FISH) analyses revealed that the depletion of the Nup93 sub-complex (but not Nup98) disengages the HOXA gene locus from the nuclear periphery, suggesting a potential role for Nup93 in tethering and repressing the HOXA gene cluster. Consistently, Nup93 knockdown increased active histone marks (H3K9ac), decreased repressive histone marks (H3K27me3) on the HOXA1 promoter and increased transcription elongation marks (H3K36me3) within the HOXA1 gene. Moreover, the combined depletion of Nup93 and CTCF (a known organizer of HOXA gene cluster) but not Nup93 alone, significantly increased GLCCI1 gene expression levels. Taken together, this suggests a novel role for Nup93 and its interactors in repressing the HOXA gene cluster.

**Conclusions:**

This study reveals that the nucleoporin Nup93 assisted by its interactors Nup188 and Nup205 mediates the repression of HOXA gene expression.

**Electronic supplementary material:**

The online version of this article (doi:10.1186/s13072-016-0106-0) contains supplementary material, which is available to authorized users.

## Background

The nuclear pore complex (NPC) is a highly conserved protein complex, localized at the nuclear periphery and is required for import and export of proteins and RNA [1, 2]. Nucleoporins in *Saccharomyces cerevisiae*, *Drosophila melanogaster* and mammalian cells are also involved in transcriptional regulation [3–7], transcriptional memory [8–10], demarcating chromatin boundaries [11, 12], differentiation, development [4, 13–15], DNA damage repair [16, 17] and chromatin organization [18, 19]. These functions are likely to involve chromatin contacts with nucleoporins. Typically, Nups contact chromatin in either an off-pore or on-pore manner. In humans, Nup98 contacts chromatin in an off-pore manner inside the nucleoplasm away from the nuclear periphery [13, 20]. In *Drosophila*, mobile Nups such as Nup98, Sec13 and Nup50 re-localize to the nucleoplasm and contact chromatin [21]. Nup153 and Megator (Mtor) are mobile nucleoporins that associate with ~25% of the *Drosophila* genome at Nucleoporin-associated regions (NARs) [22]. In neural progenitor cells, nucleoporins contact chromatin in an on-pore manner, for instance a group of genes that include GRIK1, NRG1 and MAP2 are specifically associated with Nup98 at the nuclear envelope upon transcriptional activation [13]. The yeast Nup170p associates with the RSC chromatin remodeling complex and the silencing factor Sir4p which cooperatively mediates the association of telomeres with the nuclear envelope resulting in sub-telomeric gene silencing [23]. Taken together, these studies suggest an association of nucleoporins with chromatin. However, the molecular mechanisms of nucleoporins and their interaction with chromatin in transcription regulation remain unclear. Nucleoporins in addition to their primary role in nuclear transport also function in chromatin organization. However, the mechanisms of chromatin organization mediated by stable and on-pore nucleoporins remain unclear.

The nucleoporin Nup93 sub-complex is composed of Nup93, Nup188, Nup205, Nup155 and Nup53 [24–28]. Nup93 is a highly stable nucleoporin with a relatively low dissociation rate from the nuclear pore complex (*K*_off_: 4.0 ± 3.4 × 10^−6^ s^−1^) [29]. Interestingly, ChIP-chip studies using tilling microarrays for human chromosomes 5, 7 and 16 in HeLa cells reveal that Nup93 contacts chromatin sub-domains on these chromosomes [30]. These studies show an association of Nup93 with the promoters of HOXA1, HOXA3 and HOXA5 on human chromosome 7. However, the potential role of nucleoporins in regulating HOX gene expression is unclear. This raises the intriguing possibility of Nup93 to function as an additional modulator of the HOXA chromatin sub-cluster and therefore HOXA gene expression during differentiation.

The HOXA gene locus (Chr.7p15.3) spans ~150 kb of the sub-genomic region (27,112,593–27,254,038 bp, hg19 assembly) and encodes for 11 transcription factors that are involved in pattern formation in early development [31]. Aberrant HOXA expression levels correlate with cancers and is dysregulated in breast carcinoma, human cutaneous melanoma and oral cancers [32–37]. Chromosome conformation capture studies have shown that the repressed HOXA gene cluster adopts a compact chromatin state organized as “multiple chromatin loops” for instance in undifferentiated NT2/D1 cells [38–40]. These loops of the HOXA gene loci are disrupted by the combined action of retinoic acid treatment and depletion of CTCF or PRC2 that transcriptionally activate HOXA gene expression [38].

Brown et al. [7] showed for the first time that Nup93 contacts chromatin and associates with the HOXA promoter. We extended this study to address the consequences of depleting Nup93 on HOXA gene expression. We show that Nup93 associates with and represses HOXA gene expression in a manner dependent on its interacting partners—Nup188 and Nup205, in diploid colorectal cancer cells (DLD1). The depletion of Nup93 or its interacting partners—Nup188 and Nup205, derepresses the HOXA gene cluster since this showed a marked increase in HOXA gene expression, facilitated by enhanced levels of the active histone marks (H3K9ac) and decreased levels of repressive histone marks (H3K27me3) on the HOXA1 promoter. In addition, transcription elongation marks (H3K36me3) were enriched within the HOXA1 gene. This is consistent with an untethering of the HOXA gene cluster from the nuclear periphery upon depletion of Nup93 or its interactors Nup188 or Nup205, but not in Nup98-depleted cells. Furthermore, Nup93 represses HOXA gene cluster independent of its key regulator, i.e., CTCF [38]. Taken together, this study reveals that Nup93 along with its interacting partners—Nup188 and Nup205, represses HOXA gene expression.

## Results

**Fig. 1:**
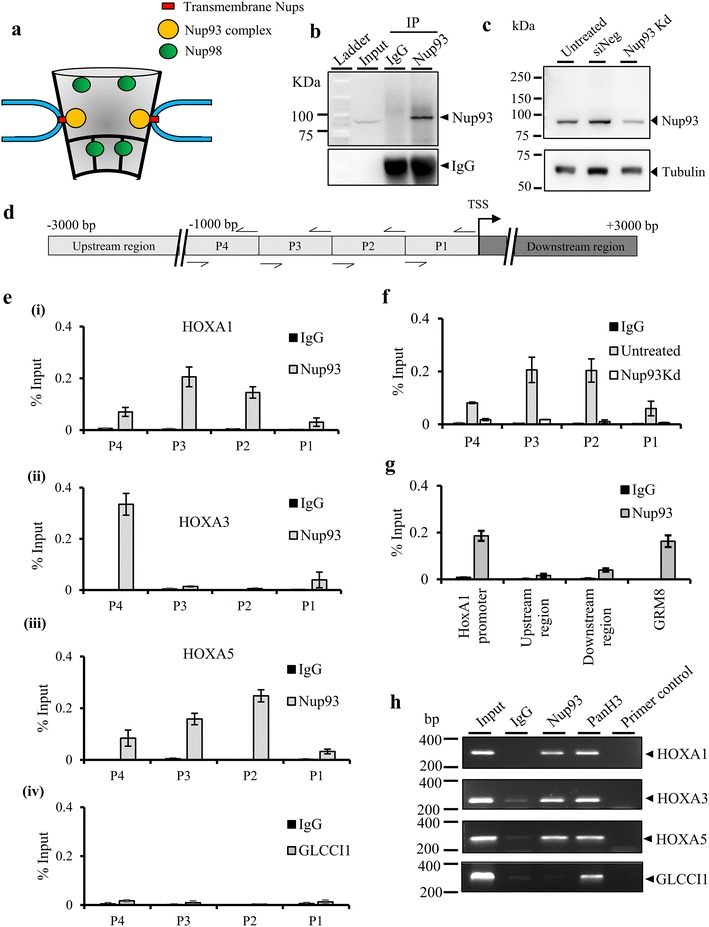
Nup93 associates with HOXA1, HOXA3 and HOXA5 promoters. **a** Pictorial representation of the nuclear pore complex (NPC) showing the relative location of the Nup93 sub-complex, and Nup98 within the nuclear pore complex [103]. **b** Immunoprecipitation of Nup93 using anti-Nup93 antibody on whole-cell extracts of DLD1 cells (a representative full blot from three independent biological replicates, *N* = 3). Anti-rabbit heavy chain IgG shows equal precipitation efficiency in IgG and Nup93 lanes. **c** A representative full Western blot showing siRNA-mediated depletion of Nup93 in DLD1 cells (*lane* Nup93 Kd), *Untreated* Untreated cells, *siNeg* ON-TARGETplus non-targeting siRNA control (a representative blot from three independent biological replicates, *N* = 3). **d** Pictorial representation of primer pair positions (P1–P4) on the promoter of HOXA1, HOXA3 and HOXA5 genes, respectively, upstream region and downstream region indicates primer position outside the HOXA1 promoter, *arrow* indicates transcription start site (TSS). **e** ChIP experiments were performed using antibodies specific to Nup93 and IgG control. Nup93 ChIP-qPCR on (*i*) HOXA1 (*ii*) HOXA3 (*iii*) HOXA5 and (*iv*) GLCCI1 promoters, respectively. *Y*-axis: immunoprecipitated DNA relative to 1% input, corrected for ChIP using non-specific IgG (*N* = 2, data from two independent biological replicates that include a total of six technical replicates), *error bar* standard error of mean (SEM). **f** Nup93 ChIP-qPCR was performed in untreated and Nup93 knockdown cells for HOXA1 promoter using primer pairs P1–P4. **g** Nup93 ChIP-qPCR using primer pairs outside HOXA1 promoter regions (upstream region and downstream region) and primers for a Nup93-associated gene (GRM8), used as a positive control. **h** ChIP-PCR amplification of HOXA1, HOXA3 and HOXA5. GLCCI1 used as negative control. Nup93 binds to ~300–600 bp on each of these HOXA promoters, (*N* = 2, representative data from two independent biological replicates)

It is well established that chromatin is associated with nuclear landmarks such as the nuclear lamina, nucleolus, nuclear bodies and the nuclear pore complex [41, 42]. However, the specific structural and functional role of these nuclear landmarks in regulating chromatin organization and gene expression remains elusive [42, 43]. As shown previously across biological systems, chromatin at the nuclear periphery is directly or indirectly associated with nuclear pore proteins [44]. Nup93—a scaffold nucleoporin at the core of the nuclear pore complex, contacts chromatin sub-domains of human chromosomes 5, 7 and 16 in HeLa cells as revealed by ChIP-chip studies using tilling microarrays for these chromosomes (Fig. 1a) [30]. Furthermore, chromosome 7 is predominantly localized toward the nuclear periphery [45]. HOXA is an important gene cluster that maps to human chromosome 7 with Nup93 binding sites on HOXA1, HOXA3 and HOXA5 gene promoters [30]. The focus of our study was to examine the consequences of depleting Nup93 and its interactors on HOXA gene expression in diploid colorectal cancer cells (DLD1). The DLD1 cell line is a colorectal adenocarcinoma cell line which maintains a stable and near diploid karyotype with a modal number of 46 chromosomes.

### Nup93 associates with HOXA promoter regions

Chromatin immunoprecipitation on chip in HeLa cells showed that Nup93 binds to the promoter regions [~1000 bp upstream of the transcription start site (TSS)] of HOXA1, HOXA3 and HOXA5 on human Chr.7p15.2 [30]. To validate the antibody that we used against Nup93, we performed immunoprecipitation assays, which detected a single band at ~93 kDa (Fig. 1b). Furthermore, siRNA-mediated knockdown followed by immunoblotting, showed a >70% depletion of Nup93 (Fig. 1c), thereby validating this antibody for ChIP experiments. In order to ascertain if Nup93 associates with the HOXA promoter region and to extend earlier observations, we performed ChIP-qPCR of specific subregions of the HOXA promoter [30] (Fig. 1d). We found that Nup93 was indeed enriched on specific subregions of HOXA1, HOXA3 and HOXA5 promoters (Fig. 1d, e), which further validated that Nup93 associates with the HOXA1, HOXA3, and HOXA5 promoter sequences, respectively (Fig. 1e). Nup93 was not enriched on GLCCI1 promoter—a gene locus ~19 Mb upstream of HOXA1 on Chr.7p21.3, and therefore served as a negative control (Fig. 1e-iv) [30]. To further validate the association of Nup93 with HOXA promoter regions, we performed ChIP-qPCR with Nup93 in Nup93-depleted cells. ChIP-qPCR results showed a significantly reduced association of Nup93 with the HOXA1 promoter upon Nup93 knockdown (Fig. 1f). To ascertain the binding preferences of Nup93, we examined whether Nup93 associates with regions outside the HOXA1 promoter (~3 Kbp upstream and downstream of the HOXA1 promoter region) (Fig. 1d) and another Nup93 target gene (GRM8) (Chr.7q31.11). Nup93 associates within the GRM8 gene body but not with its promoter [30]. We found that Nup93 does not associate with sites just outside the HOXA1 promoter (~3 Kbp) (Fig. 1g). In contrast, Nup93 was significantly enriched within the GRM8 gene (Fig. 1g). We reconfirmed our results by performing ChIP-PCR using primer pairs P3, P4, P2 and P4 for HOXA1, HOXA3, HOXA5 and GLCCI1 respectively (Fig. 1h).  In summary, these results corroborate the association of Nup93 with the HOXA1, HOXA3 and HOXA5 promoter regions.

### Nup93 requires its interactors Nup188 and Nup205 to associate with the HOXA1 promoter

**Fig. 2:**
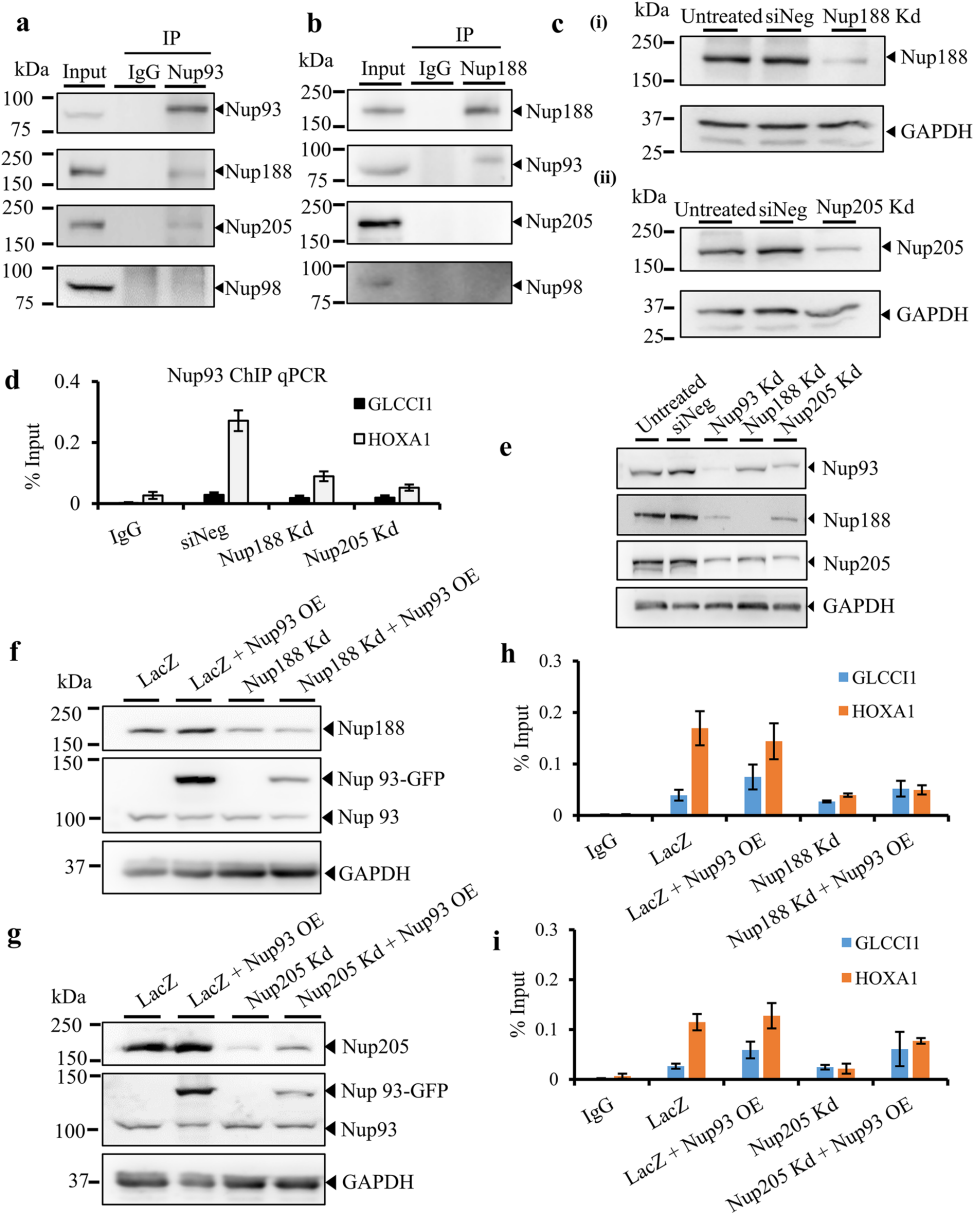
Nup93 interacts with Nup188 and Nup205 and associates with the HOXA1 promoter. **a**, **b** Immunoprecipitation was performed using antibodies specific for (**a**) Nup93; (**b**) Nup188 and IgG followed by Western blotting for Nup93, Nup188, Nup205 and negative control—Nup98 (representative data from three independent biological replicates, *N* = 3, single experiment for Nup98). **c** (*i*) Nup188 and (*ii*) Nup205 were knocked down in DLD1 cells using siRNA. A representative Western blot showing the extent of knockdown (representative Western blot from three independent biological replicates, *N* = 3). **d** ChIP experiment was performed using an anti-Nup93 antibody in untreated, non-targeting siRNA control (siNeg), Nup188 Kd (Knockdown) and Nup205 Kd cells. ChIP-qPCR analysis was used to determine the extent of Nup93 association with the HOXA1 promoter in Nup188 and Nup205 knockdown cells (Input and PanH3 in Fig. 2d are from Nup205 Kd sample) *Y*-axis: immunoprecipitated DNA relative to 1% input, corrected for ChIP using non-specific IgG (*N* = 2, data from two independent biological replicates that include a total of six technical replicates), *error bar*: standard error of mean (SEM). **e** A representative Western blot showing the effect of Nup93, Nup188 and Nup205 depletion on one another (three independent biological replicates, *N* = 3), **f**, **g** a representative Western blot showing overexpression of Nup93 upon Nup188 (**f**) and Nup205 knockdown (**g**). GAPDH was used as a loading control. **h**, **i** ChIP-qPCR was performed upon overexpression of (**f**) Nup93 in Nup188- and (**g**) Nup205-depleted cells. *Y*-axis: immunoprecipitated DNA relative to 1% input, corrected for ChIP using non-specific IgG (*N* = 2, data from two independent biological replicates that include a total of six technical replicates), *error bar*: standard error of mean (SEM)

We sought to determine whether the interactors of Nup93, i.e., Nup188 and Nup205 [46] are required for Nup93 to associate with the HOXA1 promoter region. We ascertained if Nup93 interacts with Nup188 and Nup205. Co-immunoprecipitation of Nup93 from whole-cell extracts of DLD1 cells showed that Nup93 interacts with Nup188 and Nup205, respectively (Fig. 2a, b) [46, 47]. However, Nup93 does not associate with Nup98 and neither did we detect an association between Nup188 and Nup98 (Fig. 2a, b), consistent with previous findings that Nup98 does not interact with Nup93 [46]. Reverse co-immunoprecipitation showed that Nup188 associates with Nup93 but not with Nup205 or Nup98 (Fig. 2b). Furthermore, Co-IP studies showed a reduced interaction of Nup93 with Nup188 in Nup205-depleted cells, suggesting the requirement for Nup205 in the interaction between Nup93 and Nup188 (Additional file 1: Fig. S1a). Taken together, these results suggest that Nup93 associates with Nup188 and Nup205 consistent with co-immunoprecipitation assays performed in *C. elegans* and *S. cerevisiae*, which show that Nup93 interacts with nucleoporins—Nup188 and Nup205 [26, 27, 46–48].

We determined whether the interactors of Nup93, i.e., Nup188 and Nup205 are required for Nup93 to associate with the HOXA1 promoter region (Fig. 2d). We performed ChIP with Nup93, in a background of Nup188- or Nup205-depleted DLD1 cells (Fig. 2c). ChIP-PCR and chip-qPCR performed on HOXA1 promoter region (Region P3, Fig. 1d, e–i) showed that Nup188 and Nup205 depletion decreased the occupancy of Nup93 by ~70% on the HOXA1 promoter (Fig. 2d and Additional file 1: Fig. S1b). However, the occupancy of the core histone H3 (ChIP with anti-PanH3 antibody that detects core histone H3) was unaltered on either HOXA1 or GLCCI1 promoters upon Nup188 or Nup205 depletion (Additional file 1: Fig. S1b–c). Although independent knockdowns of Nup93, Nup188 and Nup205 did not affect the transcript levels of one another (Additional file 1: Fig. S1d), we detected a decrease in their relative protein levels in an interdependent manner (Fig. 2e and Additional file 1: Fig. S1f–g). We therefore considered the possibility that the reduced occupancy of Nup93 on HOXA1 could be attributed to reduced levels of Nup93 in cells depleted of Nup188 or Nup205. To account for the decrease in the levels of Nup93 upon Nup188 or Nup205 depletion, we overexpressed Nup93 in a background of Nup188 or Nup205 depletion (Fig. 2f, g). Despite Nup93 overexpression (Fig. 2f, g, Additional file 1: Fig. S1e), Nup93 showed a reduced occupancy on the HOXA1 promoter in either Nup188- or Nup205-depleted cells (Fig. 2h, i). This suggests that overexpressed Nup93 is unable to associate with the HOXA1 promoter in the absence of Nup188 or Nup205. Taken together, these results suggest that a stable complex of Nup188–Nup93–Nup205 is required for Nup93 to associate with the HOXA1 promoter.

### HOXA gene expression is upregulated in Nup93-, Nup188- or Nup205-depleted cells

**Fig. 3:**
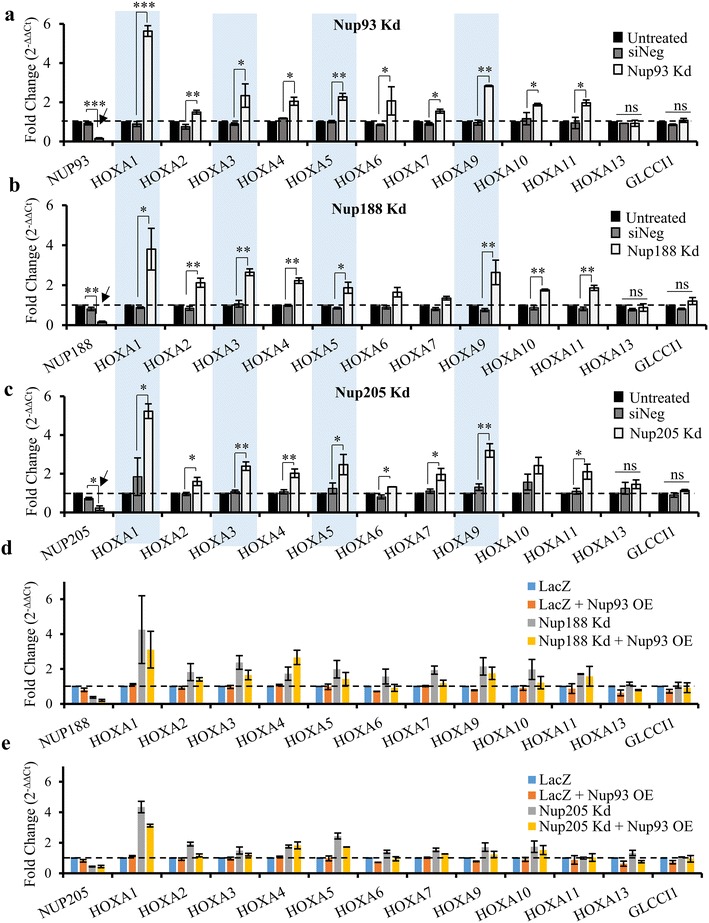
Depletion of Nup93, Nup188 and Nup205 derepresses HOXA gene cluster. **a**–**c** qRT-PCR analyses was used to determine mRNA levels of all HOXA genes (HOXA1 to HOXA13) upon (**a**) Nup93, (**b**) Nup188 and (**c**) Nup205 knockdowns in DLD1 cells. *Graph* represents fold change ($2^{{ - {\Delta \Delta }C_{\text{t}} }}$) in levels of mRNA normalized to untreated cells. Error bars: SEM, data from three independent biological replicates that include total of nine technical replicates, *p < 0.05; **p < 0.01; ***p < 0.001 (Students *t* test between siNeg and knockdown). GLCCI, served as a negative control. **d** ,**e** qRT-PCR analyses was used to determine mRNA levels of all HOXA genes (HOXA1 to HOXA13) upon Nup93 overexpression in (**d**) Nup188- and (**e**) Nup205-depleted cells. *Graph* represents fold change ($2^{{ - {\Delta \Delta }C_{\text{t}} }}$) in levels of mRNA normalized to untreated cells. *Error bars*: SEM, data from two independent biological replicates that include total of six technical replicates. GLCCI, served as a negative control

The finding that Nup93 associates with the promoters of HOXA1, HOXA3 and HOXA5 genes assisted by Nup188 and Nup205 (Figs. 1e, 2d) prompted us to investigate whether Nup93 and its interactors regulate HOXA gene expression. We independently knocked down Nup93, Nup188 and Nup205 in DLD1 cells and assessed expression levels of all genes within the HOXA gene cluster (HOXA1–HOXA13) by qRT-PCR (Fig. 3a–c). Nup93, Nup188 and Nup205 knockdown showed a >80% reduction in their transcript levels (Fig. 3a–c, arrow). Remarkably, the transcript levels of HOXA genes (HOXA1, HOXA3, HOXA5 and HOXA9) were strikingly upregulated (fold change >twofold) upon Nup93 Kd (Fig. 3a). HOXA1 showed an increase in transcript levels in all three nucleoporin knockdowns to ~four–sixfold, suggesting a significantly greater impact on HOXA1 expression levels upon Nup93, Nup188 and Nup205 knockdowns. Furthermore, HOXA1, HOXA3, HOXA5 and HOXA9 were significantly upregulated in Nup188- and Nup205-depleted cells (Fig. 3b, c). Interestingly, the expression levels of HOXA13 and GLCCI1 were unaffected in all the three Nup knockdowns (HOXA13, GLCCI1, Fig. 3a–c). We also used two independent siRNA oligonucleotides to knockdown Nup93 (Additional file 2: Fig. S2a), which showed an upregulation of HOXA1, HOXA3, HOXA5 and HOXA9 genes but not of HOXA13 or GLCCI1 (Fig. S2b), consistent with previous results (Fig. 3a). Of note, the depletion of Nup98 did not alter gene expression levels of the HOXA gene cluster and GLCC1 (Additional file 2: Fig. S2b), further suggesting a novel role for Nup93 and its interacting partners—Nup188 and Nup205, in regulating HOXA gene expression in DLD1 cells.

Since ChIP showed a reduced occupancy of Nup93 on the HOXA1 promoter despite Nup93 overexpression in Nup188- and Nup205-depleted cells (Fig. 2h, i), we determined the effect of Nup93 overexpression on HOXA transcript levels in Nup188- and Nup205-depleted cells, respectively. Interestingly, Nup93 overexpression was incapable of rescuing HOXA gene expression in Nup188- and Nup205-depleted cells (Fig. 3d, e, Additional file 1: Fig. S1e). Taken together, these assays further reiterate a requirement for Nup188 and Nup205 in Nup93-mediated repression of HOXA gene expression.

We were curious to examine the consequences of allowing cells to recover after Nup93 depletion and asked whether the upregulation of HOXA gene expression was reversible or irreversible, since an irreversible upregulation of HOXA may imply a feedback effect to sustain HOXA upregulation. We allowed cells to recover in culture for 8 days after 48 h of Nup93, Nup188 and Nup205 knockdown in DLD1 cells. Interestingly, we detected a significant restoration in the transcript levels of Nup93, Nup188 and Nup205, respectively (Additional file 2: Fig. S2c–e) accompanied by the downregulation of HOXA1 and HOX9 transcript levels, while HOXA13 levels remained unaltered (Additional file 2: Fig. S2c–e). Taken together, these results uncover a novel role for Nup93 and its interacting partners in the repression of the HOXA gene cluster.

### HOXA gene loci is untethered from the nuclear periphery in Nup93-, Nup188- and Nup205-depleted cells

**Fig. 4:**
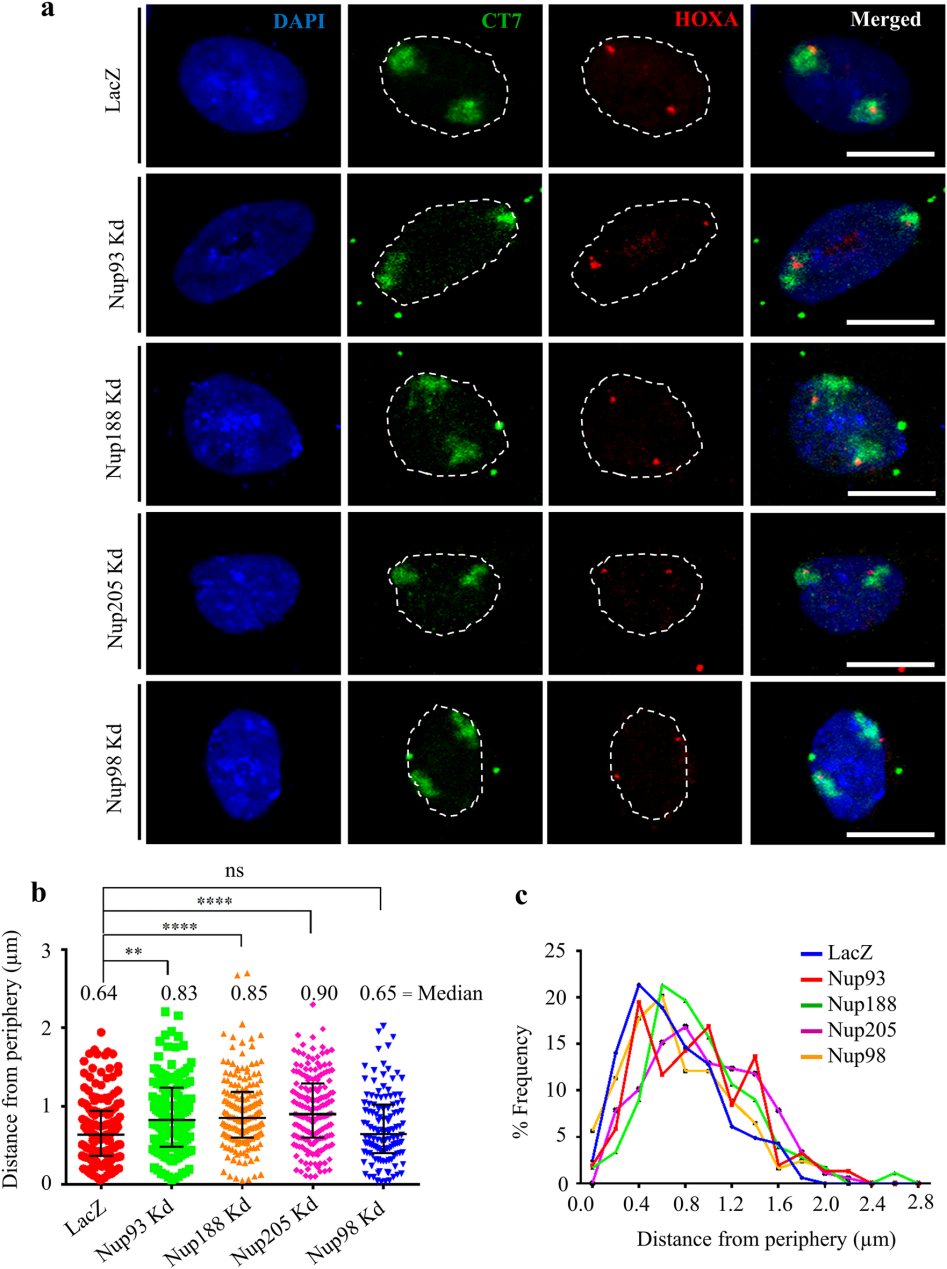
HOXA gene loci is untethered from the nuclear periphery upon Nup93, Nup188 and Nup205 depletion. **a** Representative images (maximum intensity projection of a confocal image stack) of 3D-FISH for HOXA (*red*), CT7 (*green*) and DAPI (*blue*) performed on siLacZ-, Nup93-, Nup188-, Nup205- and Nup98-depleted DLD1 cells. *Scale bar* ~10 μm, *white dotted line* indicates nuclear boundary. **b** *Dot scatter plot* showing shortest distance of HOXA gene locus from nuclear periphery demarcated by DAPI in siLacZ (*n* = 164 loci signals)-, Nup93 (*n* = 154)-, Nup188 (*n* = 178)-, Nup205 (*n* = 178)- and Nup98 (*n* = 124)-depleted DLD1 cells, *horizontal bar* represents median with interquartile range. Data from two independent biological replicates, **p < 0.01; ****p < 0.001 (Kolmogorov–Smirnov test). **c** % Frequency distribution profile of HOXA gene locus from nuclear periphery plotted as bins of ~0.2 µm each from the nuclear periphery. *Y*-axis represents % frequency of HOXA locus pooled from two independent biological replicates

The significant upregulation of HOXA genes upon Nup93, Nup188 and Nup205 depletion prompted us to determine the spatial localization of HOXA gene locus upon Nup93, Nup188 or Nup205 depletion in the interphase nucleus. Gene loci tethered to the nuclear periphery are typically maintained in a state of repression [49]. We performed three-dimensional fluorescence in situ hybridization (3D-FISH) followed by confocal imaging of fluorescently labeled HOXA gene locus and Chromosome 7 Territories (CT7) in cells independently depleted of Nup93, Nup188 and Nup205, respectively (Fig. 4a). We measured the shortest distance of the HOXA gene locus from the nuclear periphery [50]. HOXA gene loci were predominantly localized closer to the nuclear periphery in control cells [median = 0.64 µm from the edge of the nucleus in control cells (Fig. 4b, c)]. Interestingly, Nup93-, Nup188- or Nup205-depleted cells showed an ~0.2-µm shift of the HOXA gene loci away from the nuclear edge (Fig. 4b, c). In contrast, Nup98 depletion did not affect the positioning of the HOXA loci with respect to the nuclear periphery (Fig. 4b, c). Taken together, this suggests that Nup93 and its interactors are likely to tether the HOXA gene locus closer to the nuclear periphery in order to maintain HOXA gene locus in a repressed configuration, while depletion of Nup93 or either of its interacting partners results in a movement of the HOXA gene locus toward the nuclear interior (Fig. 4c).

### Nup93 depletion alters the relative occupancy of histone marks on the HOXA1 promoter

**Fig. 5:**
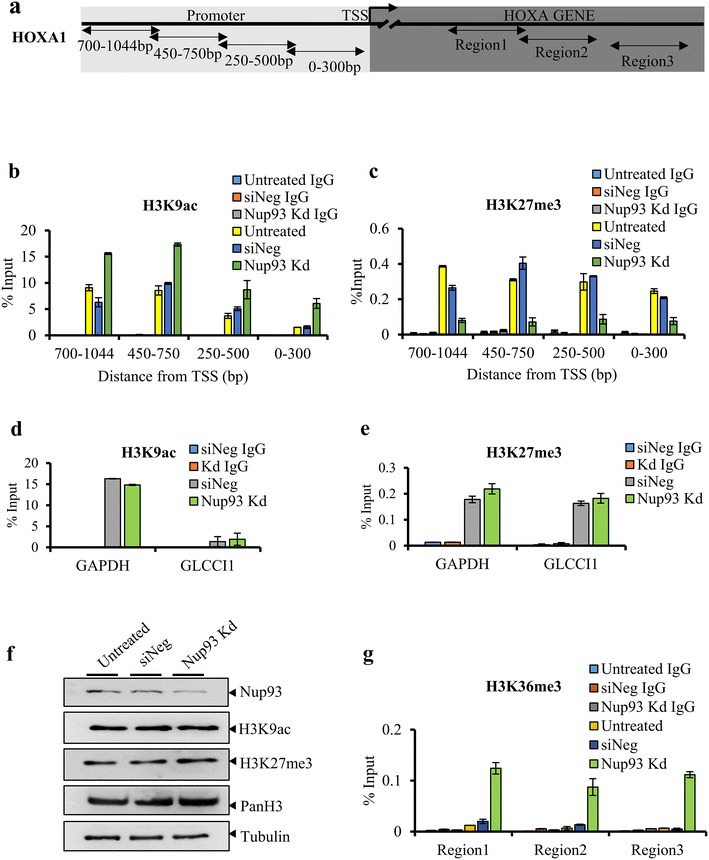
Nup93 depletion alters the occupancy of histone marks on HOXA1 promoter. **a** Pictorial representation of the HOXA1 promoter and regions within the HOXA1 gene (Region 1–Region 3). *Left* (*light gray*) promoter of HOXA1 gene, *double arrowheads*: overlapping primer positions on HOXA1 promoter. *Right* (*dark gray*) regions within HOXA1 gene, *double arrowheads*: ChIP-qPCR primer positions within HOXA1 gene (Region 1–Region 3). **b**, **c** ChIP experiments were performed using antibodies specific to (**b**) H3K9ac, (**c**) H3K27me3 and IgG in untreated, siNeg and Nup93 knockdown cells (IgG is below detection limit <0.2% of input in ‘b–d’, re-plotted in Additional file 2: Fig. S2h–j), **d**, **e** GAPDH promoter and GLCCI1 promoter were used as positive and negative controls, respectively. *Y*-axis: immunoprecipitated DNA is relative to 1% input, corrected for ChIP using non-specific IgG (data from two independent biological replicates that include a total of six technical replicates), *error bars*: SEM. **f** Representative Western blot of untreated, siNeg and Nup93 Kd cells showing that total levels of H3K9ac and H3K27me3 are unaltered. PanH3 and Tubulin were used as loading controls (data from a single experiment). **g** Elongation mark (H3K36me3) shows increased occupancy on the HOXA1 gene (Region 1, Region 2 and Region 3), data from two independent biological replicates that include a total of six technical replicates, *error bars*: SEM, Nup93 knockdown alters occupancy of histone marks on HOXA1 promoter

We determined whether the observed derepression of the HOXA gene cluster upon Nup93 depletion is associated with an altered occupancy of active and inactive histone marks on the HOXA1 promoter region (Fig. 5a). We performed ChIP with active (H3K9ac) and repressive (H3K27me3) histone marks on the HOXA1 promoter in Nup93-depleted cells (Fig. 5b–e). We determined the levels of active and repressive histone marks on the HOXA1 promoter upon Nup93 knockdown, since HOXA1 showed the highest increase in transcript levels (>four–sixfold) in cells depleted of Nup93 or its interacting partners—Nup188 or Nup205 (Fig. 3a–c). We performed ChIP with antibodies against active (H3K9ac) and repressive (H3K27me3) histone marks followed by ChIP-qPCR with overlapping primers ~1 Kb upstream of the HOXA1 transcription start site (Fig. 5a). We detected a significant enrichment of the active histone mark (H3K9ac), ~1 Kb upstream of the transcription start site of the HOXA1 gene (Fig. 5b) and a marked decrease in the occupancy of the repressive histone mark (H3K27me3) upon Nup93 knockdown (Fig. 5c). Notably, the relative levels of active and repressive histone marks were unaltered upon Nup93 depletion on the promoters of control genes—GAPDH and GLCCI1 (Fig. 5d, e). Furthermore, the total levels of H3K9ac, H3K27me3 and PanH3 were unaffected upon Nup93 depletion in DLD1 cells (Fig. 5f). Taken together, these results suggest a strong correlation between Nup93 depletion and the relative enrichment of the active histone mark and decreased occupancy of the inactive histone mark on the HOXA1 promoter. This is consistent with previous studies which show that silencing of the HOXA gene cluster is mediated by Polycomb (PcG) proteins by the recruitment of repressive histone marks (H3K27me3) on the HOXA gene promoter of NT2/D1 embryonal carcinoma cell line and in HeLa cells [38, 51].

Since we detected a striking increase in the expression levels of the HOXA1 gene upon Nup93, Nup188 and Nup205 knockdown, we asked whether HOXA1 gene expression correlates with active transcriptional elongation, i.e., active transcription of the HOXA1 gene. We performed ChIP with H3K36me3—a histone mark enriched during transcriptional elongation [52], with the HOXA1 gene (Fig. 5a). Interestingly, Nup93 knockdown showed a specific enrichment of H3K36me3 on all three regions (Region 1–Region 3) within the HOXA1 gene (Fig. 5g). Taken together, these experiments strongly suggest that active transcription of the HOXA1 gene upon depletion of Nup93 is associated with an increased occupancy of active histone marks and decreased levels of repressive histone marks.

### Nup93 depletion reduces nuclear import but does not affect nuclear export

**Fig. 6:**
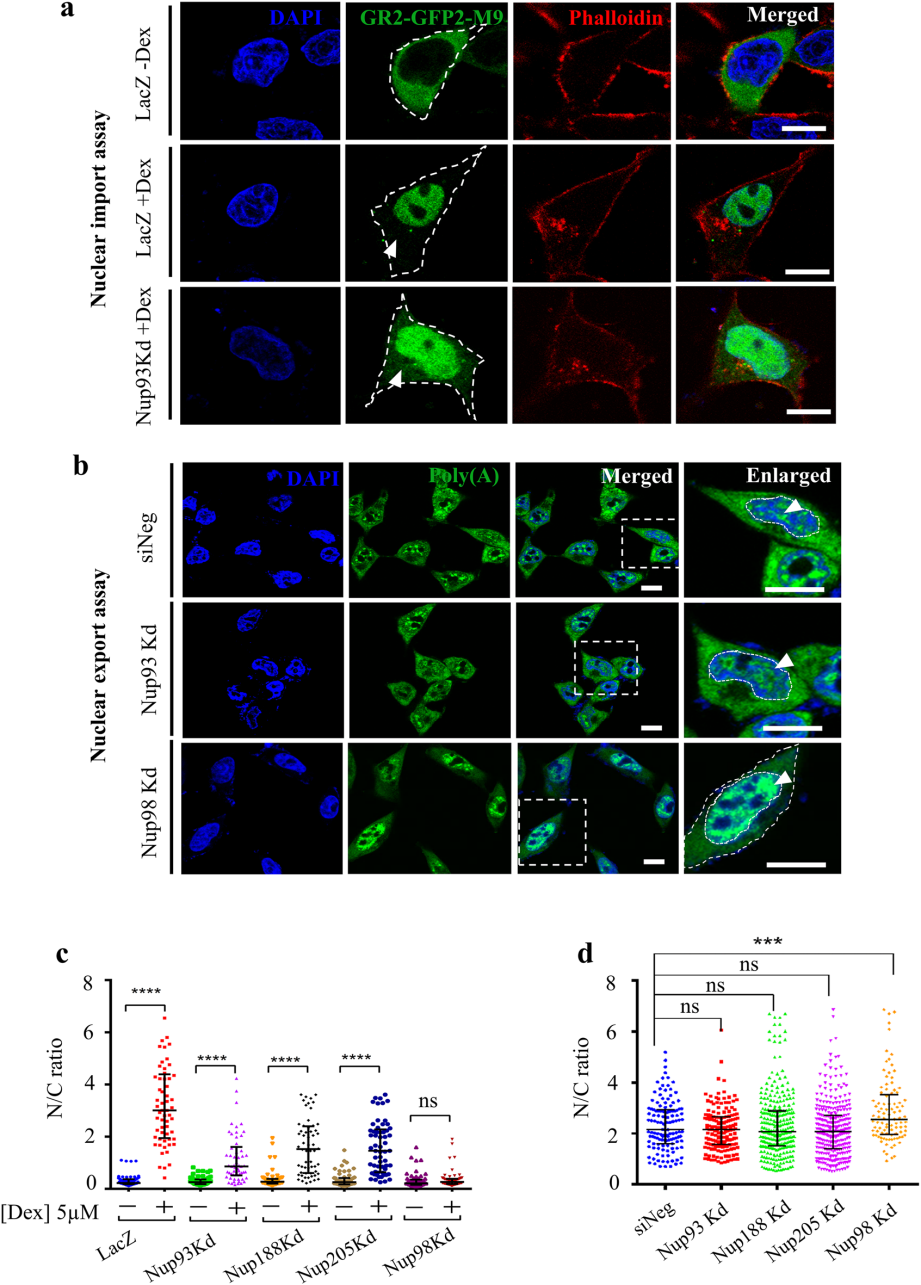
Nup93 depletion reduces nuclear import but does not affect nuclear export. **a** A representative image of nuclear import assay performed using GR2-GFP2-M9 construct transfected in cells treated independently with LacZ and siNup93. To induce nuclear import of GR2-GFP2-M9 fusion protein, cells were treated with dexamethasone (Dex) (5 µM) for 30 min, *white arrowhead* indicates absence of cytoplasmic GFP in LacZ + Dex and residual cytoplasmic GFP in Nup93 Kd + Dex. *Scale bar* ~10 µm. **b** A representative image of Poly(A) RNA FISH performed using FAM-labeled oligo(dT) probe (*green*) in siNeg, Nup93 Kd, Nup188 Kd, Nup205 Kd and Nup98 Kd, *scale bar* ~10 µm, *white arrowhead* indicates Poly(A) RNA foci in the nucleus. Nuclear boundary is marked by *dotted line* in enlarged panel. Nup98 enlarged panel shows both nuclear and cell boundary with white dotted line. **c** Nuclear/cytoplasmic (N/C) ratio of GR2-GFP2-M9 was determined by quantifying its relative fluorescence intensity in the nucleus and cytoplasm. Scatter plot of GFP signals expressed as nuclear-to-cytoplasmic ratios from LacZ (*n* = 60 cells), Nup93 Kd (*n* = 57), Nup188 Kd (*n* = 60), Nup205 Kd (*n* = 59) and Nup98 Kd (*n* = 60), data from 2 independent biological replicates (****p < 0.0001). **d** Poly(A) RNA distribution was determined by quantifying its fluorescence intensity in the nucleus and cytoplasm. Scatter plot of Poly(A) signals expressed as nuclear (N)-to-cytoplasmic (C) ratios from siNeg (*n* = 127 cells), Nup93 Kd (*n* = 158), Nup188 Kd (*n* = 288), Nup205 Kd (*n* = 288); N/C ratio was not significant (ns) when compared to siNeg (*p* > 0.05), while Nup98 Kd (*n* = 97) shows a relatively higher nuclear-to-cytoplasmic ratio (N/C ratio) of Poly(A) signals (***p = 0.0017). Two independent biological replicates for siNeg, Nup93 Kd and Nup98 Kd. Data from a single experiment for Nup188 Kd and Nup205 Kd. *Horizontal line* represents median, *p* values obtained from Mann–Whitney *U* test. Nuclear transport of Poly(A) RNA was unaffected in Nup93-, Nup188- or Nup205- depleted cells

In order to address the role of Nup93 and its interactors in HOXA gene expression, we were curious to determine whether nuclear transport was affected in cells depleted of Nup93 or either of its interactors Nup188 or Nup205. To determine the effect of Nup93 depletion on nuclear import, we transfected DLD1 cells with a dexamethasone-inducible reporter construct consisting of the hormone-responsive domain of glucocorticoid and GFP fused to the M9 core domain (GR2-GFP2-M9core) [53]. Upon transient transfection, the reporter construct was exclusively localized in the cytoplasm, which translocated to the nucleus within 30 min in the presence of 5 µM dexamethasone (glucocorticoid hormone analogue) in control cells (LacZ, Fig. 6a and c, Additional file 3: Fig. S3). Nup93, Nup188 or Nup205 depletion showed a reduced nuclear import of the reporter GFP construct as compared to control cells after 30 min of dexamethasone addition (Fig. 6a, c, Additional file 3: Fig. S3). Notably, Nup98 depletion blocked nuclear import of the reporter GFP (Fig. 6c and Additional file 3: Fig. S3). Taken together, this suggests that the depletion of Nup93 or its interactors results in reduced nuclear import.

As a readout of nuclear export, we examined the nucleocytoplasmic distribution of fluorescently labeled Poly(A) RNA in Nup93-, Nup188- or Nup205-depleted cells (Fig. 6b, d), since Poly(A) RNA is abundant in cells and is typically associated with mature transcripts [54]. Poly(A) RNA is detectable within the nucleus as foci (Fig. 6b) and diffusely within the cytoplasm (Fig. 6b, siNeg panel). We did not detect a significantly altered distribution of Poly(A) signals inside or outside the nucleus in Nup93-, Nup188- or Nup205-depleted cells, as compared to control cells (siNeg, Fig. 6b, d, Additional file 4: Fig. S4). This is consistent with a normal nuclear export in nuclear reassembly assays performed in Nup188–Nup93 immunodepleted *Xenopus* egg extracts [46]. Depletion of Nup98—an established regulator of nuclear export, showed a retention of Poly(A) RNA in the nucleus, evidenced by a significant increase in the nuclear-to-cytoplasmic ratio of Poly(A) RNA in DLD1 cells (Fig. 6b, d) [55–57]. Taken together, these assays suggest that, although nuclear export was not significantly affected, nuclear import was reduced but not inhibited in cells depleted of Nup93 or its interacting partners.

### HOXA derepression upon Nup93 depletion is independent of CTCF

**Fig. 7:**
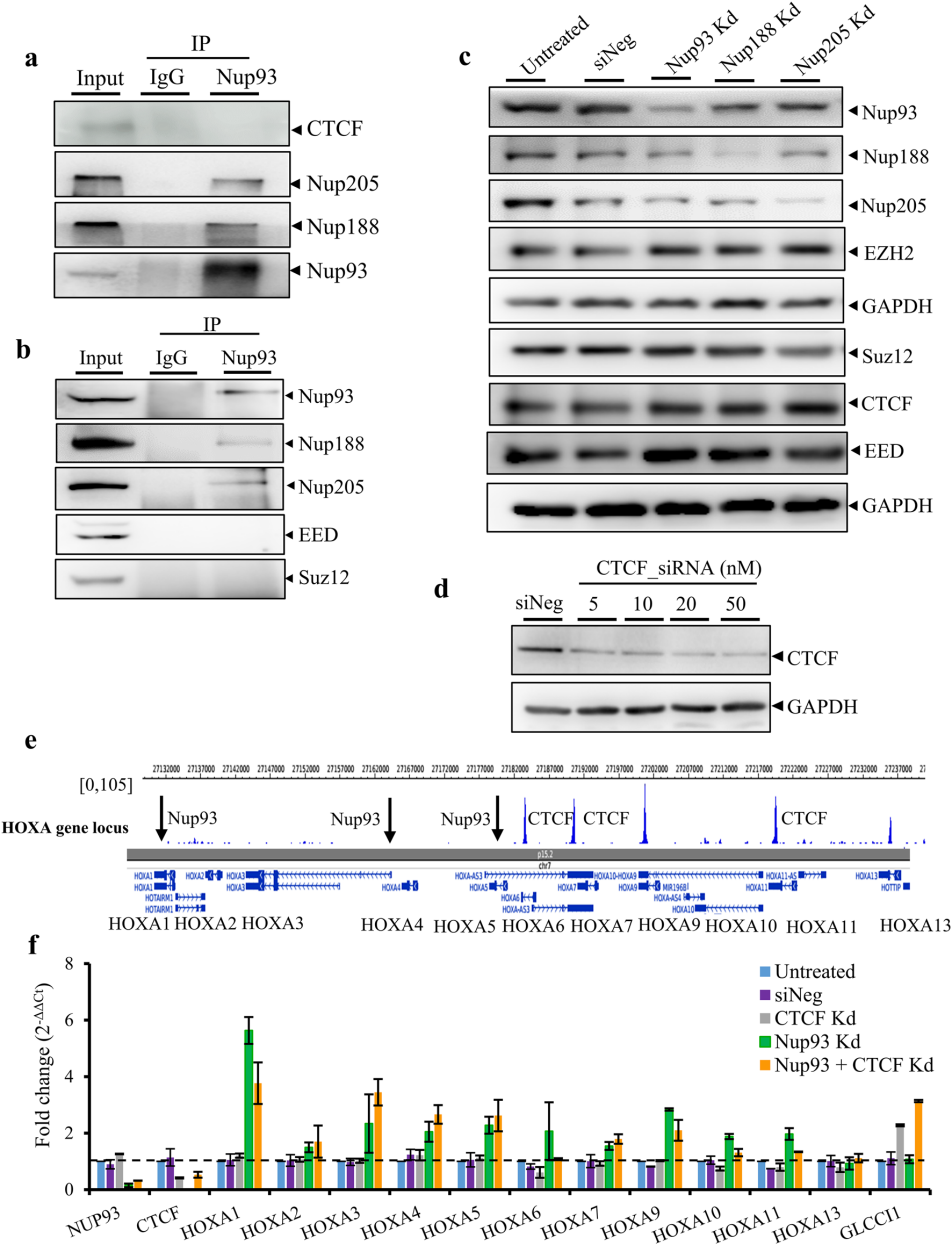
HOXA is upregulated upon Nup93 depletion independent of CTCF. **a** Co-immunoprecipitation was performed using anti-Nup93 antibody and negative control IgG followed by Western blotting for CTCF, Nup205, Nup188 and Nup93 (data from two independent biological replicates, *N* = 2), **b** Co-IP for Nup93 and Western blot for Nup93, Nup188, Nup205 (data from three independent biological replicates, *N* = 3), PRC2 complex proteins EED and Suz12 (data from a single experiment). **c** Representative Western blot showing the levels of Nup93, Nup188, Nup205, Nup98, EZH2, Suz12, CTCF, EED upon Nup93, Nup188 and Nup205 Kd (data from a single experiment). **d** Representative Western blot showing siRNA-mediated knockdown of CTCF in DLD1 cells. **e** Epigenome Browser view of CTCF (GSM749729) (*arrow* indicates potential binding sites of Nup93) on HOXA gene cluster. **f** qRT-PCR analysis was used to determine mRNA levels of all HOXA genes (HOXA1 to HOXA13) upon CTCF and combined Nup93 + CTCF knockdowns in DLD1 cells. *Graph* represents fold change ($2^{{ - {\Delta \Delta }C_{\text{t}} }}$) in levels of mRNA normalized to untreated cells. *Error bars* SEM, data from two independent biological replicates (*N* = 2) that includes total of 6 technical replicates, GLCCI, served as control. Nup93 Kd data (*green bars*) is from Fig. 3a, plotted here for comparison between Nup93 Kd with Nup93 + CTCF Kd (*orange bars*). Nup93 does not interact with CTCF or PRC2 complex proteins. Nup93 Kd upregulates HOXA gene expression independent of CTCF. CTCF depletion alone upregulates GLCCI1, which is unaffected upon Nup93 knockdown

CTCF and PRC2 complex proteins regulate chromatin looping and expression of the HOXA gene cluster in embryonic stem cells [31, 38, 39, 51, 58–60]. We sought to ask whether CTCF regulates HOXA gene expression levels. We determined whether CTCF and PRC2 complex proteins associate with Nup93 (Fig. 7a, b). Co-immunoprecipitation (Co-IP) assays did not reveal an association between Nup93 and CTCF (Fig. 7a), or between Nup93 and PRC2 complex proteins (EED or Suz12) in DLD1 cells (Fig. 7b). Furthermore, levels of CTCF or the PRC2 complex proteins (EZH2, Suz12 and EED) were unaltered in Nup93-, Nup188- or Nup205-depleted cells (Fig. 7c). CTCF is a known organizer of the HOXA gene cluster and has conserved binding sites that are proximal to the 5’ region of the HOXA gene cluster which do not overlap with Nup93 binding sites (Fig. 7e) [38]. We therefore investigated the effect of CTCF depletion on HOXA gene expression, by knocking down CTCF alone and in combination with Nup93 (Fig. 7f). Interestingly, the depletion of CTCF alone did not affect gene expression levels of any of the genes within the HOXA gene cluster (Fig. 7f, gray bars). In contrast, CTCF depletion significantly upregulated the transcript levels of GLCCI1—a gene otherwise unaffected upon Nup93 knockdown (Fig. 7f, GLCCI1). This is consistent with the role of CTCF in regulating the organization of topologically associated domains (TADs), the disruption of which potentially impact distant genomic regions [61]. Notably, the combined knockdown of both CTCF and Nup93 upregulated expression levels of HOXA genes, comparable to Nup93 knockdown alone (Fig. 7f, compare green and orange bars). Of note, GLCCI1 showed an enhanced upregulation in Nup93 + CTCF Kd cells, suggesting an altered regulatory role for CTCF in cells subjected to a combined depletion of Nup93 and CTCF. Taken together, we conclude from these assays that Nup93 and CTCF may have complementary functions in the organization of the HOXA gene cluster.

## Discussion

The regulated expression of the HOX family of transcription factors is required during early development and differentiation, whereas its untimely expression in differentiated cells is associated with disease [33, 34, 37, 62]. HOX gene expression is also maintained in differentiated cells such as human skin fibroblasts in a manner that retains their tissue-specific origin [63]. ChIP-chip studies using tiling microarrays revealed an association of Nup93 with human chromosomes 5, 7 and 16 [30]. Nup93 was enriched on the HOXA sub-cluster of human chromosome 7 [30]. Here, we show that Nup93 associates with the HOXA gene cluster in a manner dependent on its interactors Nup188 and Nup205. Furthermore, depletion of these nucleoporins showed a significant increase in the expression levels of HOXA genes. This was consistent with a disengagement of the HOXA gene locus from the nuclear periphery in Nup93-/Nup188-/Nup205-depleted cells. In addition, the upregulation of HOXA genes upon Nup93 depletion was associated with an increase in active histone marks, reduced inactive marks and enrichment of a transcription elongation mark.

### Implications of the association of nucleoporins with chromatin

Several studies across organisms have consistently shown an association of the mobile nucleoporins such as Nup98, Nup50 and Nup153 with chromatin in addition to regulating nuclear transport [3, 6, 9, 13, 15, 22, 23]. We corroborated previous findings of Brown et al. [30] and show that Nup93 indeed associates with the promoters of HOXA1, HOXA3 and HOXA5 and represses HOXA gene expression (Fig. 3a–c). It is conceivable that the repressive mechanism of Nup93 could potentially extend to other HOX gene clusters such as the HOXB, HOXC and HOXD, respectively. Chromatin conformation capture assays (such as 5C) have shown that the silenced HOXA gene cluster adopts a folded loop structure in a human myeloid leukemia cell line THP1 [64]. We speculate that nucleoporins such as Nup93 may further modulate the local three-dimensional organization of the topologically associated domains (TADs) within the HOX gene cluster [65]. Our studies provide evidence to the growing body of literature which reinforce the role of nucleoporins in regulating chromatin organization and gene expression. Gene expression regulation is typically accompanied by an altered occupancy of active and inactive histone marks on gene promoters [66, 67]. The active state of the HOXA gene cluster is marked by active histone marks such as H3K9ac and H3K4me3, while the inactive state shows an enrichment of inactive marks such as H3K9me3 and H3K27me3 [36, 66, 68]. For instance, histone deacetylases and PRC2 complex proteins modify levels of active and inactive histone marks on the HOXA gene cluster in NT2/D1 embryonal carcinoma cells [38]. Interestingly, a recent Dam-ID study showed that Nup93 associates with chromatin at the nuclear periphery [69]. Considering the localization of the HOXA gene cluster on the gene poor chromosome 7 territory, proximal to the nuclear periphery, we found a sequestration of the HOXA gene cluster to the nuclear periphery potentially mediated by the Nup93 sub-complex but not Nup98 (Fig. 4). We surmise that the depletion of Nup93, Nup188 or Nup205 and their reduced stability, enhances the accessibility of the HOXA gene cluster to transcriptional activators and epigenetic modulators that could facilitate their untimely expression of HOXA genes—the physiological ramifications of which remain unclear.

### Role of nucleoporins in nuclear transport and chromatin organization

Nucleoporins regulate nuclear import and export of mRNA, RNA and proteins [1]. In addition, an increasing number of evidences implicate nucleoporins in gene regulation [4, 69–75]. Furthermore, the composition of the nuclear pore complex (NPC) is variable across cells types, which interestingly has limited effect on nuclear transport [14]. In embryonic stem cells, Nup210 is absent but is specifically incorporated into the NPC during differentiation [14]. Dam-ID studies reveal that Nup153 regulates expression of cell identity genes independent of its role in nuclear transport [69]. Nup98 is involved in both nucleocytoplasmic transport and gene regulation [3, 13, 76, 77], since Nup98 interacts with the mRNA export factor Rae1 and regulates mRNA export [57]. Nup98 associates with developmentally active genes such as GRIK1, ERBB4, NRG1 and DCC and regulates their expression levels during differentiation [13]. The Nup98-HOXA9 fusion protein associates with and inappropriately activates the HOX gene cluster in mouse embryonic stem cells in a manner dependent on the Crm1 protein [78]. Interestingly, Nup98 depletion in DLD1 cells did not alter either the spatial localization or the expression levels of the HOXA gene (Fig. 4 and Fig. S2b), notwithstanding its impact on nuclear transport (Fig. 6). This suggests an independent role for Nup98 in regulating nuclear transport but not HOXA gene expression. However, in cells depleted of Nup93, Nup188 or Nup205, nuclear export was relatively unaffected although nuclear import was reduced (Fig. 6a). Taken together, these findings implicate nucleoporins such as Nup93, Nup188 and Nup205 as modulators of chromatin organization in addition to their nuclear transport functions.

### Potential mechanisms of nucleoporin–chromatin interactions

The mechanisms by which core nucleoporins associate with DNA are unclear. More importantly, several findings suggest that nucleoporins are involved in chromatin remodeling owing to their association with chromatin modifiers such as the SAGA complex, HDACs, RSC complex, SUMO proteases, SENP1, SENP2 and MSL complex [23, 77, 79–82]. Chromatin remodeling complexes such as the SAGA complex—a transcriptional activator, associates with the nuclear pore complex and activates HXK1, INO1 and GAL genes when recruited to the NPC [5, 10, 83–87]. Nup2, Nup60, Nic96, Nup116, Mlp1 and Mlp2 are enriched on transcriptionally active regions in *S. cerevisiae* [85, 88]. Furthermore, ARP6 links the active housekeeping gene RPP1A, involved in ribosome biogenesis to the nuclear pore complex [89]. Nup170p represses ribosomal biogenesis genes and genes on the sub-telomeric region [23, 89]. Nup120 and Nup133 also core nucleoporins repress SUC2 gene expression in yeast [90]. Interestingly, Nup93 tethers and regulates the expression of cell identity genes, predominantly localized at the nuclear periphery [69]. The tethering of HOXA gene cluster to the nuclear periphery and its repression by the Nup93 sub-complex adds to the repertoire of nucleoporin-mediated gene repression events (Fig. 4). Analyses of protein–protein interaction networks using BIOGRID [91] of human Nup93 shows that Nup93 interacts with chromatin modifiers such as HDAC11, HDAC9, HDAC5 and PCR2 complex proteins—EED and Suz12. It is conceivable that Nup93 and its interactors associate with transcriptional repressors in repressing the HOXA gene cluster, although we did not detect a direct association between Nup93 and the chromatin repressive complex (PRC2) (Fig. 7b). ChIP-mass spectrometric approaches may identify putative interactors of Nup93 involved in chromatin organization.

### Nucleoporins as repressors of HOXA gene expression independent of CTCF

Regulation of HOXA gene expression is essential during early development, since the aberrant expression of HOX genes leads to developmental defects [92–94]. Active HOX genes cluster into transcriptionally active domains as shown using chromatin conformation capture analyses in mouse embryonic tissues [95]. Similarly, HOXA gene regulation is also important in adult tissues since their aberrant expression is associated with various cancers [34]. Furthermore, CTCF is an important regulator of the 3D organization and silencing of the HOXA gene cluster [40]. Notably, CTCF is associated closer to the 5’ region of the HOX gene cluster, in a manner that does not overlap with Nup93 binding sites, suggesting the complementary and potentially independent roles of Nup93 and CTCF in the maintenance of HOXA gene repression. However, it is unclear if CTCF silences HOXA gene cluster in differentiated cells by recruiting regulatory proteins such as PRC2. We showed that Nup93 depletion in DLD1 cells did not alter the levels of CTCF or PRC2 complex proteins—EZH2, Suz12 and EED (Fig. 7c). We surmise that CTCF or PRC2 proteins may have altered chromatin occupancy in the absence of Nup93 sub-complex, which remains to be elucidated by ChIP-sequencing of CTCF or PRC2 complex proteins in the absence of Nup93 [38]. This is further consistent with the significant upregulation of the GLCCI1 gene in cells depleted of both Nup93 and CTCF as compared CTCF-depleted cells (Fig. 7f).

We speculate a novel role for Nup93 and its interactors in regulating chromatin compaction of the HOXA gene cluster in a mechanism that curtails the aberrant expression of HOXA genes. Our studies open up challenging new frontiers for identifying the structural and molecular mechanisms of nucleoporin-mediated chromatin organization and function in paradigms of development, differentiation and disease.

## Conclusions

Our studies unravel a novel role for nucleoporins Nup93 and its interactors Nup188 and Nup205 in mediating the repression and tethering of the HOXA gene cluster to the nuclear periphery in diploid DLD1 cells. Depletion of Nup93, Nup188 or Nup205 significantly enhances HOXA gene expression. The elevated levels of HOXA gene expression upon the depletion of Nup93 or its interactors—Nup188 and Nup205, is associated with an increase in the occupancy of active histone marks, and decreased levels of inactive histone marks with a concomitant increase in transcriptional elongation marks within the HOXA gene.

## Methods

### Cell culture

Colorectal adenocarcinoma cell line—DLD1, was a gift from the laboratory of Thomas Ried. DLD1 cells were cultured in RPMI media (Invitrogen, 11875), supplemented with 10% fetal bovine serum (FBS, Invitrogen, 6140-079, Carlsbad, USA), 100 U/ml penicillin/100 µg/ml of streptomycin at 37 °C with 5% CO_2_. The authenticity of DLD1 cells was validated independently by karyotyping (data not shown).

### Transient siRNA-mediated knockdown and overexpression

Transient knockdowns were performed using siRNA oligonucleotides from Dharmacon, USA. Briefly, DLD1 cells (~0.2 × 10^6^) were plated in individual wells of a six-well plate, 24 h prior to transfection for cells to attain a confluency of ~50–60%. The cells were transfected with siRNA oligonucleotides (50 nM) using RNAiMax (Invitrogen). After 48 h of knockdown, cells were processed for Western blots or RNA extraction. For recovery experiments, cells were allowed to recover in culture for 8 days after 48 h of Nup93, Nup188 and Nup205 depletion. Nup93 knockdown was performed by using a combination of oligo-1 (5′GCGCTAATTTACTACTGCA3′) and oligo-2 (5’AGAGTGAAGTGGCGGACAA3′)—25 nM each. Two independent siRNA oligos against Nup188, Nup205 and Nup98 were tested for their knockdown efficiency. Nup188 oligo-1 (5′GGUAGUAGGCAGACCAAUAUU3′), oligo_2 (5′GCCTTTCTGCGCTTGATCACCACCC3′); Nup205 oligo-1 (5′GGAAUUAAUCCCAGAACUAUU3′), oligo2-(5′AGAUGGUGAAGGAGGAAUAUU3′); Nup98 oligo-1 (5′GTGAAGGGCTAAATAGGAA3′), oligo-2 (5′TGTCAGACCCTAAGAAGAA3′). CTCF knockdown was performed using a single oligo (5′CAAGAAGCGGAGAGGACGA3′) at a final concentration of 50 nM. ON-TARGETplus non-targeting siRNA (siNeg, Dharmacon) was used as a negative control.

For overexpression experiments, cells were first transfected with siRNA against Nup188 and Nup205 using Lipofectamine RNAi-Max reagent (Invitrogen). After 24 h, cells were washed with DPBS and transfected with full-length Nup93 (Nup93-pEGFP-N1) using Mirus2020 reagent (cat. MIR5400). After 48 h of Nup93-GPF transfection, cells were either fixed with 4% paraformaldehyde or harvested for RNA extraction using Trizol. Nup93-GFP construct was a kind gift from Radha Chauhan.

### Protein extraction and immunoblotting

Cells were lysed in RIPA buffer (50 mM Tris–Cl pH 7.4, 150 mM NaCl, 0.1% SDS, 0.1% sodium azide, 0.5% sodium deoxycholate, 1 mM EDTA, 1% NP-40, 1× protease inhibitor cocktail) and centrifuged at 13,000*g* for 10 min at 4 °C. The supernatant was separated and used for protein estimation using BCA kit (cat no. 23225). Total protein (20 µg, estimated to be within the linear range of detection) was used for each sample preparation. Samples were lysed in 1× Laemmli buffer (Tris–HCl pH 6.8, 2% SDS, 20% glycerol, 0.2% bromophenol blue, 0.025% β-mercaptoethanol) and denatured at 95 °C for 5 min. Protein samples were resolved by SDS-PAGE and were transferred to activated polyvinylidene fluoride membrane (PVDF, Millipore, cat no. IPVH00010), followed by blocking with 5% nonfat-dried skim milk/1× TBST (Tris-buffer saline, 0.1% Tween 20) for 1 h at RT. Primary antibodies were diluted in 0.5% milk/1× TBST buffer. All antibody dilutions are within the linear range of detection. Rabbit anti-Nup93 (1:500, sc-292099, Lot-E0211, Santa Cruz Biotechnology, CA), rabbit anti-Nup188 (1:1000, Abcam, ab86601, Lot-GR43443-4), mouse anti-Nup98 (1:500, sc-74553, Lot-H0108, Santa Cruz Biotechnology, Santa Cruz, CA), rabbit anti-Nup205 antibody (1:500, HPA024574, Lot-R11937, Atlas Antibodies), rabbit anti-EED (1:500, ab4469, Lot-GR51357-1), rabbit anti-EZH2 (1:500, ab3748, Lot-GR252135-1), rabbit anti-Suz12 (1:500, ab12073, Lot-GR79631-1) and rabbit anti-CTCF antibody (1:500, 07-729, Lot-2375606, Millipore). Secondary antibodies: donkey anti-rabbit immunoglobulin G horseradish peroxidase (1:10,000, GE NA9340V) and sheep anti-mouse immunoglobulin G-HRP (1: 10,000, NA9310V) were diluted in 0.5% milk (1× TBST). The blots were developed using enhanced chemiluminescence detection reagents (ECL Prime, 89168-782) at incremental exposures of 10 s acquired under a chemiluminescence system LAS4000 (GE). Densitometry analysis of Western blots was done using ImageJ software from three independent biological replicates. GAPDH was used as internal control for normalization.

### Reverse transcription-PCR and real-time quantitative PCR

Cells were washed with 1X PBS, and total RNA was extracted using Trizol method [96]; cDNA was synthesized from total RNA with the ImProm-II reverse transcription system using Oligo(dT) primers (Promega, A3800); cDNA was used as a template and RT-PCR was carried out using intron–exon junction primers (Additional file 5: Table S1). β-actin and glyceraldehyde-3-phosphate dehydrogenase (GAPDH) were used as internal controls. The template cDNA was serially diluted to optimize the extent of amplification in the linear range. Real-time quantitative PCR was performed using Bio-Rad RT-PCR instrument (CFX96 Touch) in 10 µl of reaction mixtures containing KAPA SYBR Green RT-PCR mix and 2 µM each of the forward and reverse primer, respectively (Additional file 5: Table S2). Fold change was calculated by double normalization of Ct values to the internal control and untreated samples by $2^{{ - {\Delta \Delta }C_{\text{t}} }}$ method [97].

### Immunoprecipitation (IP) and Co-IP

Cells were lysed using IP lysis buffer (50 mM HEPES—pH 8.0, 140 mM NaCl, 1% NP-40, 0.1% sodium deoxycholate, 0.1% SDS and 1 mM EDTA) in the presence of 1X complete protease inhibitor cocktail (PIC). Lysates were pre-cleared using protein A dynabeads (Invitrogen, 10002D), 1 h at 4 °C. Pre-cleared extracts were incubated overnight at 4 °C with anti-Nup93, anti-Nup205 and anti-Nup188 antibodies independently (2 μg/500 μg of total protein). IP complexes were captured using protein A dynabeads (pre-blocked with 0.5% BSA/1X PBS) and washed with lysis buffer and high salt wash buffer-1 (500 mM NaCl in lysis buffer), and wash buffer-2 (20 mM Tris–HCL (pH 8.0), 1 mM EDTA, 0.5% NP-40 and 50× PIC). Elution was performed using Laemmli loading buffer and analyzed by Western blotting. For Co-IP, cells were lysed in Co-IP buffer (50 mM Tris–HCl, pH 8.0, 150 mM NaCl and 0.5% NP-40) supplemented with protease inhibitor cocktail. Co-IP washes were performed in the same Co-IP lysis buffer.

### Chromatin immunoprecipitation

DLD1 cells (~1.0 × 10^7^) were cross-linked using 1% formaldehyde for 10 min at RT. Cross-linking was quenched using 150 mM glycine, and cells were lysed in 1 ml swelling buffer (25 mM HEPES pH 8.0, 1.5 mM MgCl_2_, 10 mM KCl, 0.1% NP-40, 1X PIC), and nuclei were recovered by centrifugation at 2000 rpm. Fixed nuclei were re-suspended in 1 ml sonication buffer (50 mM HEPES pH 8.0, 140 mM NaCl, 1 mM EDTA, 1% Triton-X-100, 0.1% sodium deoxycholate and 0.1% SDS) supplemented with protease inhibitor cocktail and sonicated using Bioruptor Twin sonicator (Diagenode) to generate fragment sizes of ~100–500 bp. Sonicated chromatin was separated by centrifugation at 13,000*g* at 4 °C for 10 min. Supernatant was pre-cleared using protein A dynabeads (Invitrogen), 1 h at 4 °C. The amount of DNA was estimated using NanoDrop 2000. Nup93 antibody validated according to ENCODE guidelines (~2 µg) was added to ~100 µg of chromatin sample and diluted to ~1 ml in sonication buffer and incubated overnight at 4 °C [98]. IP complexes were captured using protein A dynabeads (pre-blocked with 0.5% BSA/1X PBS) and washed three times (at 4 °C, 11 rpm on end-to-end rotor) each with sonication buffer, wash buffer-1 (50 mM HEPES pH 8.0, 500 mM NaCl, 1 mM EDTA, 1% Triton X-100, 0.1% sodium deoxycholate and 0.1% SDS), wash buffer-2 (20 mM Tris–HCL pH 8.0, 1 mM EDTA, 0.5% NP-40, 250 mM LiCl, 0.5% sodium deoxycholate and 1× PIC) and TE buffer. Immunoprecipitated chromatin was eluted twice in 200 µl of elution buffer (50 mM Tris-HCL pH 8.0, 1 mM EDTA, 1% SDS, 50 mM NaHCO_3_) at 65 °C for 10 min. Input and IP fractions were treated with 20 µg RNase A for 1 h at 42 °C followed by 40 µg Proteinase K for 1 h at 65 °C. Reverse cross-linking was performed overnight at 65 °C. DNA was extracted with phenol/chloroform/isoamyl alcohol (25:24:1), ethanol precipitated using 3 M sodium acetate (pH 5.2) and 2 µg of glycogen. DNA samples were washed with 70% ethanol and re-suspended in 10 µl of nuclease free water. ChIP-PCR was performed using primers listed in Additional file 5: Table S1. DNA was quantified by real-time qPCR, and fold enrichment over input was calculated by % input method from $2^{{ - {\Delta \Delta }C_{\text{t}} }}$ [97, 99]. All ChIP-qPCR experiments were performed in two independent biological replicates as recommended in the ENCODE guidelines [100].

### Three-dimensional fluorescence in situ hybridization (3D-FISH)

3D-FISH assays were performed as described previously [101]. DLD1 cells (~0.2 × 10^6^) were seeded on coverslips in a six-well plate. After 48 h of Nup93 knockdown, the cells were washed with ice-cold 1× PBS and treated with cytoskeletal (CSK) digestion buffer (0.1 M NaCl, 0.3 M sucrose, 3 mM MgCl_2_, 10 mM PIPES (pH 7.4), 0.5% Triton X-100) for 5 min followed by fixation with 4% PFA in 1X PBS (pH 7.4) for 10 min at RT. The cells were permeabilized in 0.5% Triton X-100 (prepared in 1× PBS) for 10 min and incubated in 20% glycerol (prepared in 1× PBS) for 60 min followed by four freeze–thaw cycles in liquid nitrogen. The cells were washed three times in 1× PBS and treated with 0.1 N HCl for 10 min followed by three washes in 1× PBS for 5 min each. The cells were incubated in 50% formamide (FA)/2 × saline sodium citrate (SSC) (pH 7.4) overnight at 4 °C or until used for hybridization. Cells were hybridized with 3 µl of human whole chromosome 7 paint (Applied Spectral Imaging (ASI), Israel, or MetaSystems, USA) and nick-translated BAC DNA probe (RP11-1132K14) for HOXA gene locus (3 µl). Post-hybridization, coverslips were washed in 50% FA/2× SSC (pH 7.4), thrice for 5 min each at 45 °C, followed by three washes for 5 min each in 0.1× SSC at 60 °C. Coverslips were then counterstained with DAPI for 2 min, washed in 2× SSC and mounted in Slowfade Gold antifade (Invitrogen S36937). Image acquisition was performed on a Leica SP8 confocal microscope with a 63X Plan-Apo1.4 NA oil immersion objective using scan zoom of 2.5. Acquisition of Z-stacked images (voxel size of 0.105 μm  ×  0.105 μm  ×  0.30 μm) was at 512  ×  512 pixels per frame using 8-bit pixel depth for each channel. The line averaging was set to 4, and images were collected sequentially in a three-channel mode. Distances of gene loci from the nuclear periphery were measured (in μm) in 3D using the boundary of the DAPI signal as a marker of the nuclear periphery [50]. Briefly, surface rendering was performed for the nucleus (blue channel), HOXA gene locus (red channel), CT7 (green channel), using Huygens Professional software.

### Nuclear import assay

Nuclear import assay was performed as described previously [53]. DLD1 cells (~0.2 × 10^6^) were seeded on coverslips in a six-well plate. After 24 h of siRNA transfection, cells were transfected with the vector encoding GR2-GFP2-M9core fusion protein. After 48 h of transfection, cells were treated with 5 μM dexamethasone (Sigma) for 30 min at 37 °C and fixed with 4% paraformaldehyde. Cells were permeabilized with 0.5% Triton X-100 and immunostained with Phalloidin-Alexa 594 (Invitrogen, cat. A12381) to mark the cell boundary. Finally, cells were stained with DAPI and mounted in antifade solution. Images were acquired using Leica SP8 confocal microscopy using a 63× objective/N.A. 1.4 using 405, 488 and 594 nm and lasers, zoom set to 2.0. The mean fluorescence intensity of the GFP signal was determined for each cell (demarcated by phalloidin) and nucleus (demarcated by DAPI), and expressed as a ratio of the nuclear to cytoplasmic fluorescence intensity using ImageJ. Nuclear/cytoplasmic (N/C) fluorescence intensity ratios were calculated and plotted using GraphPad Prism software. Statistical analysis was performed using Mann–Whitney test. The GR2-GFP2-M9 construct was a kind gift from Ralph Kehlenbach.

### Poly(A) fluorescence in situ hybridization (FISH)

DLD1 cells (~0.2 × 10^6^) were seeded on coverslips in a six-well plate. After 48 h of Nup93 knockdown, the cells were fixed with 4% PFA in 1× PBS (pH 7.4) for 15 min at RT. Cells were re-fixed and permeabilized with chilled methanol for 5 min, followed by incubation in 2× SSC at RT for 10 min. Cells were hybridized with 100 µl of hybridization mix (40% formamide, 10% dextran sulfate, 0.1 mg salmon sperm DNA and 5 ng/ml of FAM oligo dT prepared in 2× SSC solution) at 37 °C for 3 h. Coverslips were washed twice with 2× SSC, followed by washes with 0.1× SSC. Cells were stained with DAPI and mounted in antifade solution. Images were acquired using confocal microscopy using a 63× objective/N.A. 1.4 using 488 and 405 nm lasers, zoom set to 1.0. The mean fluorescence intensity of the FISH signal was determined for each cell and nucleus (demarcated by DAPI), and expressed as a ratio of the nuclear to cytoplasmic fluorescence intensity using the Cell Profiler software [102]. Nuclear/cytoplasmic (N/C) fluorescence intensity ratios were calculated and plotted using GraphPad Prism software. Statistical analysis was performed using Mann–Whitney test.

### Data accession from WashU Epigenome Browser

WashU Epigenome Browser was used to visualize the openly available CTCF (GSM749729) ChIP-seq data from HeLa cells.

*Antibodies used*: All antibodies and their dilutions used in this study are provided in Additional file 5: Table S3.

## Additional files

**Additional file 1: Figure S1.** **a** Co-Immunoprecipitation of Nup93 upon depletion of Nup205, shows reduced interaction between Nup93 and Nup188. **b** Nup93 ChIP PCR experiment was performed in Nup188 and Nup205 depleted cells, a representative gel image from 2 independent biological replicates. **c** ChIP qPCR for control PanH3 in siNeg, Nup188 and Nup205Kd cells, Y-axis represents immunoprecipitated DNA relative to 1% input (N = 2, independent biological replicates). **d** qRT-PCR analyses was performed upon Nup93, Nup188 and Nup205 depletion to check their relative transcript levels. Y-axis indicates fold change in levels of mRNA normalized to untreated cells. Error bars: S.E.M, data from a single experiment that includes 3 technical replicates. **e** qRT-PCR analyses upon Nup93 over expression in a background of Nup188 or Nup205 depletion as indicated. Y-axis indicates fold change in levels of mRNA normalized to untreated cells. Error bars: S.E.M, data from two biological replicates that includes 6 technical replicates. **f** Western blot quantification of Fig. 2e from 3 independent biological replicates. Y-axis: relative band intensity quantified using Image-J. **g** Ponceaue staining for western blot represented in Fig. 2e.

**Additional file 2: Figure S2.** **a** Two independent siRNA oligos were used to knockdown Nup93, Nup188, Nup205 and Nup98.A representative western blot form two independent biological replicates. GAPDH was used as an internal control. **b** qRT-PCR was performed for entire HOXA cluster upon Nup93, Nup188, Nup205 and Nup98 depletion using two independent siRNA oligos. Y-Axis represent fold change normalized to untreated. Data from two independent biological replicates, Error bars: S.E.M. **c**–**e** Effect of allowing cells to recover for 8 days after 48 hours of knockdown of (**c**) Nup93; (**d**) Nup188 and (**e**) Nup205, qRT-PCR analysis was used to determine mRNA levels of HOXA1, HOXA9 and HOXA13 genes after 48 h knockdown and 8 days of recovery. Error bars-S.E.M, data from one biological replicate that includes 3 technical replicates. Nup93, Nup188 and Nup205 recover to comparable levels as that of untreated cells after 8 days, along with a concomitant repression of HOXA1, HOXA9 and HOXA13 transcript levels. **h–j** IgG levels in untreated, siNeg and Nup93 Kd replotted from Fig. 4b–d, since they are below the detection limit.

**Additional file 3: Figure S3.** Representative images of nuclear import assay performed using GR2-GFP2-M9 construct transfected in control (LacZ) and Nup93, Nup188, Nup205 and Nup98 depleted cells upon—and +Dex (dexamethasone) treatment (to induce nuclear import of GR2-GFP2-M9 fusion protein cells were treated with 5 µM dexamethasone for 30 min).

**Additional file 4: Figure S4.** Representative images of Poly(A) RNA FISH performed using FAM labeled oligo(dT) probe (green) in Nup188 Kd and Nup205 Kd. Scale bar: ~10 µm.

**Additional file 5: Table S1.** List of ChIP-qPCR primers. **Table S2.** List of RT-PCR primers. **Table S3.** Antibodies and their dilutions used in this study.

## Abbreviations

Kd: knockdown
IP: immunoprecipitation
ChIP: chromatin immunoprecipitation
FISH: fluorescence in situ hybridization
HDAC: histone deacetylase
PRC: Polycomb repressive complex
qPCR: quantitative polymerase chain reaction
H3K9ac: histone H3 lysine 9 acetylation
H3K27me3: histone H3 lysine 27 tri-methylation
H3K36me3: histone H3 lysine 36 tri-methylation
ENCODE: encyclopedia of DNA elements
