# Supplementary figures and images for: HOXA repression is mediated by nucleoporin Nup93 assisted by its interactors Nup188 and Nup205
Source: Epigenetics Chromatin. 2016 Dec 3;9:54. doi: 10.1186/s13072-016-0106-0 (PMC5135769; doi:10.1186/s13072-016-0106-0)

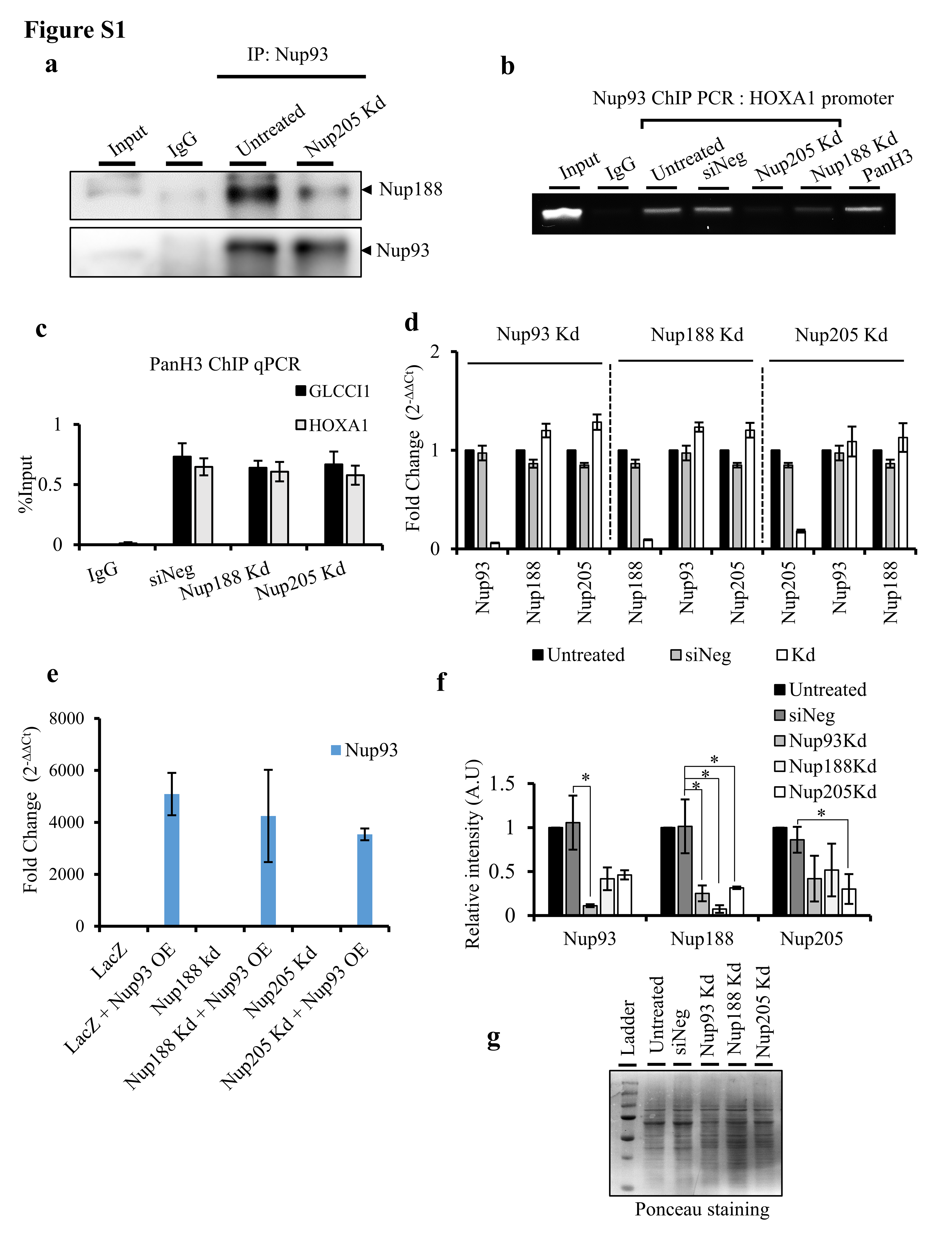

Supplement: Supplementary file 1 — Additional file 1: Figure S1. a Co-Immunoprecipitation of Nup93 upon depletion of Nup205, shows reduced interaction between Nup93 and Nup188. b Nup93 ChIP PCR experiment was performed in Nup188 and Nup205 depleted cells, a representative gel image from 2 independent biological replicates. c ChIP qPCR for control PanH3 in siNeg, Nup188 and Nup205Kd cells, Y-axis represents immunoprecipitated DNA relative to 1% input (N = 2, independent biological replicates). d qRT-PCR analyses was performed upon Nup93, Nup188 and Nup205 depletion to check their relative transcript levels. Y-axis indicates fold change in levels of mRNA normalized to untreated cells. Error bars: S.E.M, data from a single experiment that includes 3 technical replicates. e qRT-PCR analyses upon Nup93 over expression in a background of Nup188 or Nup205 depletion as indicated. Y-axis indicates fold change in levels of mRNA normalized to untreated cells. Error bars: S.E.M, data from two biological replicates that includes 6 technical replicates. f Western blot quantification of Fig. 2e from 3 independent biological replicates. Y-axis: relative band intensity quantified using Image-J. g Ponceaue staining for western blot represented in Fig. 2e. [file 13072_2016_106_MOESM1_ESM.tif]

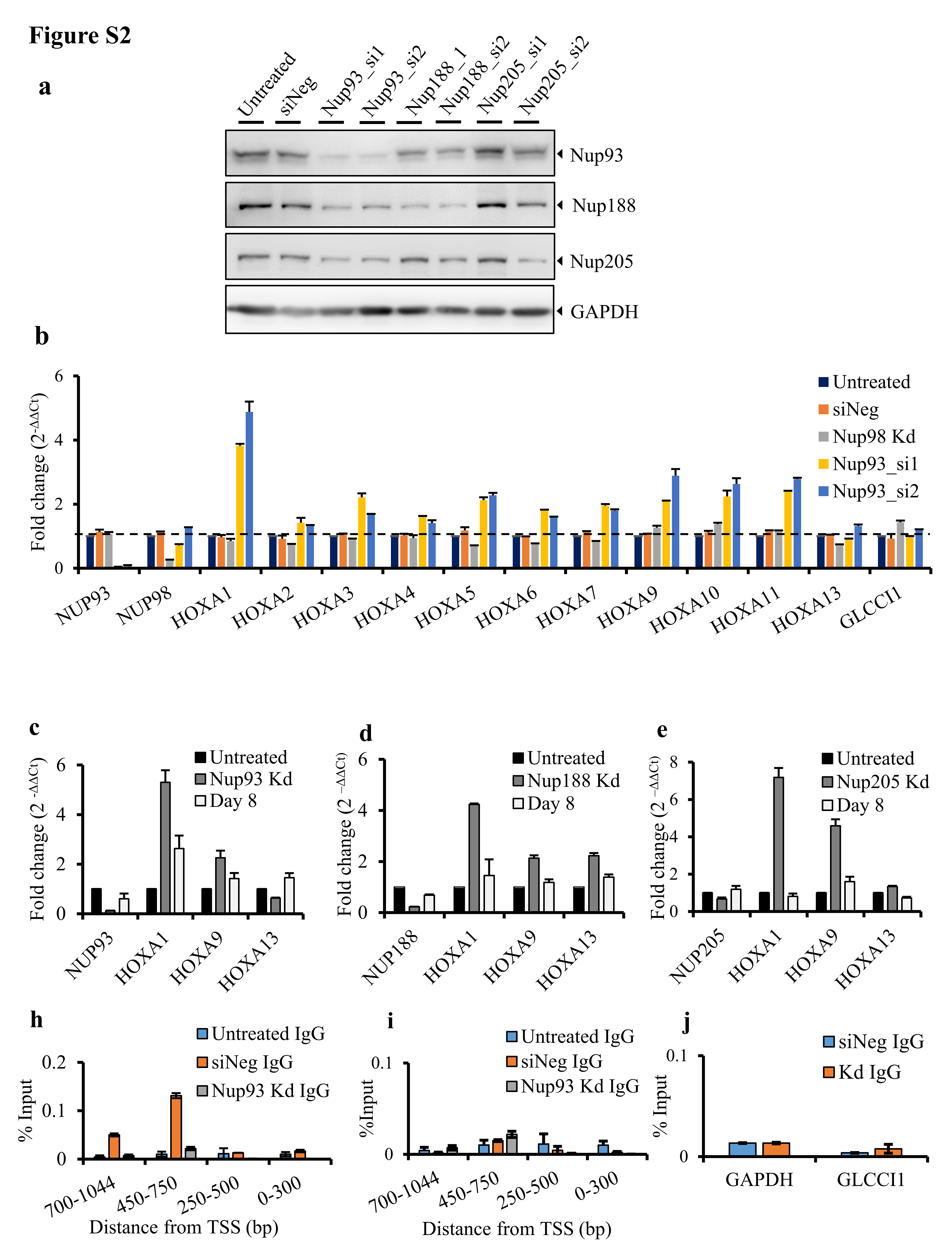

Supplement: Supplementary file 2 — Additional file 2: Figure S2. a Two independent siRNA oligos were used to knockdown Nup93, Nup188, Nup205 and Nup98.A representative western blot form two independent biological replicates. GAPDH was used as an internal control. b qRT-PCR was performed for entire HOXA cluster upon Nup93, Nup188, Nup205 and Nup98 depletion using two independent siRNA oligos. Y-Axis represent fold change normalized to untreated. Data from two independent biological replicates, Error bars: S.E.M. c–e Effect of allowing cells to recover for 8 days after 48 hours of knockdown of (c) Nup93; (d) Nup188 and (e) Nup205, qRT-PCR analysis was used to determine mRNA levels of HOXA1, HOXA9 and HOXA13 genes after 48 h knockdown and 8 days of recovery. Error bars-S.E.M, data from one biological replicate that includes 3 technical replicates. Nup93, Nup188 and Nup205 recover to comparable levels as that of untreated cells after 8 days, along with a concomitant repression of HOXA1, HOXA9 and HOXA13 transcript levels. h–j IgG levels in untreated, siNeg and Nup93 Kd replotted from Fig. 4b–d, since they are below the detection limit. [file 13072_2016_106_MOESM2_ESM.tif]

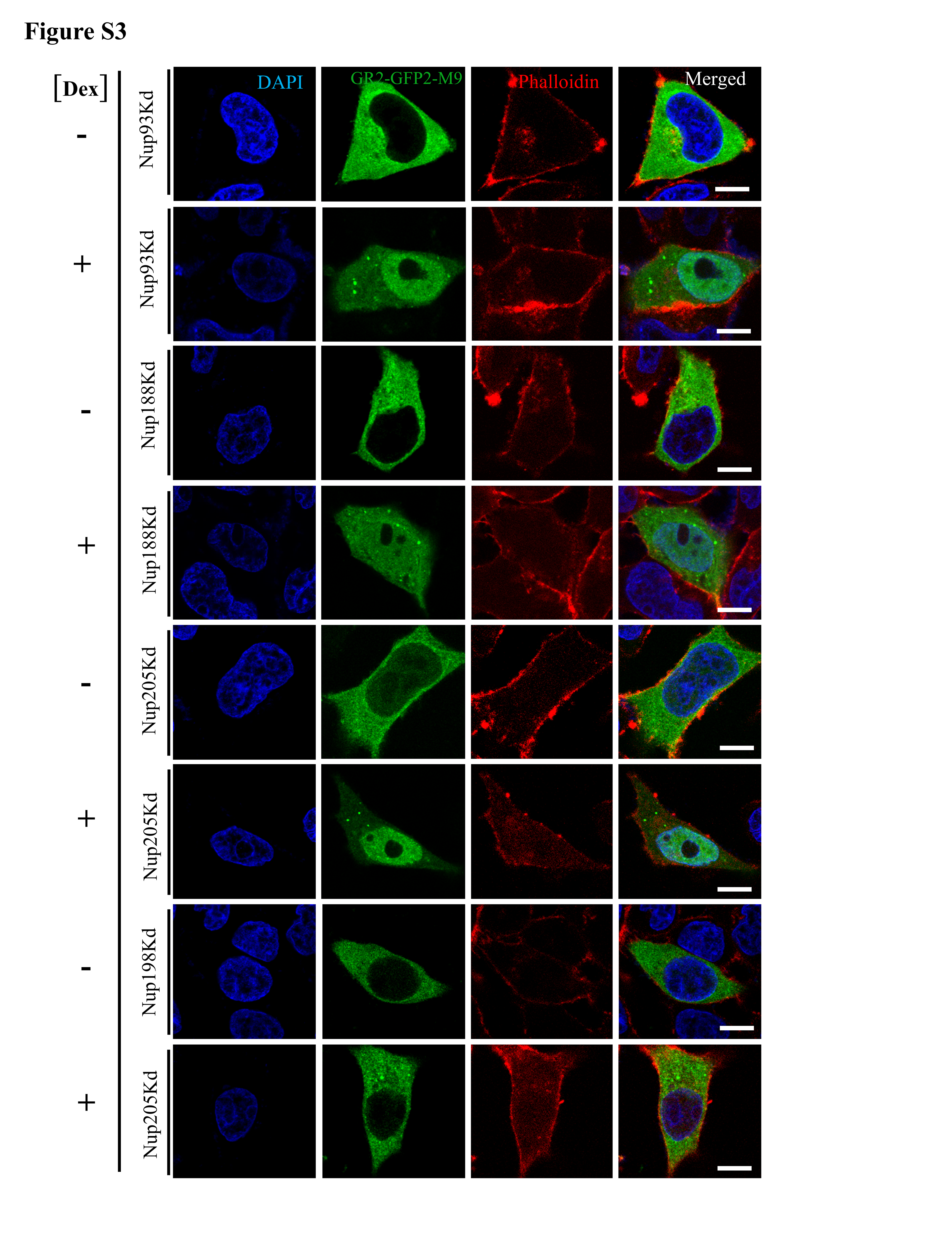

Supplement: Supplementary file 3 — Additional file 3: Figure S3. Representative images of nuclear import assay performed using GR2-GFP2-M9 construct transfected in control (LacZ) and Nup93, Nup188, Nup205 and Nup98 depleted cells upon—and +Dex (dexamethasone) treatment (to induce nuclear import of GR2-GFP2-M9 fusion protein cells were treated with 5 µM dexamethasone for 30 min). [file 13072_2016_106_MOESM3_ESM.tif]

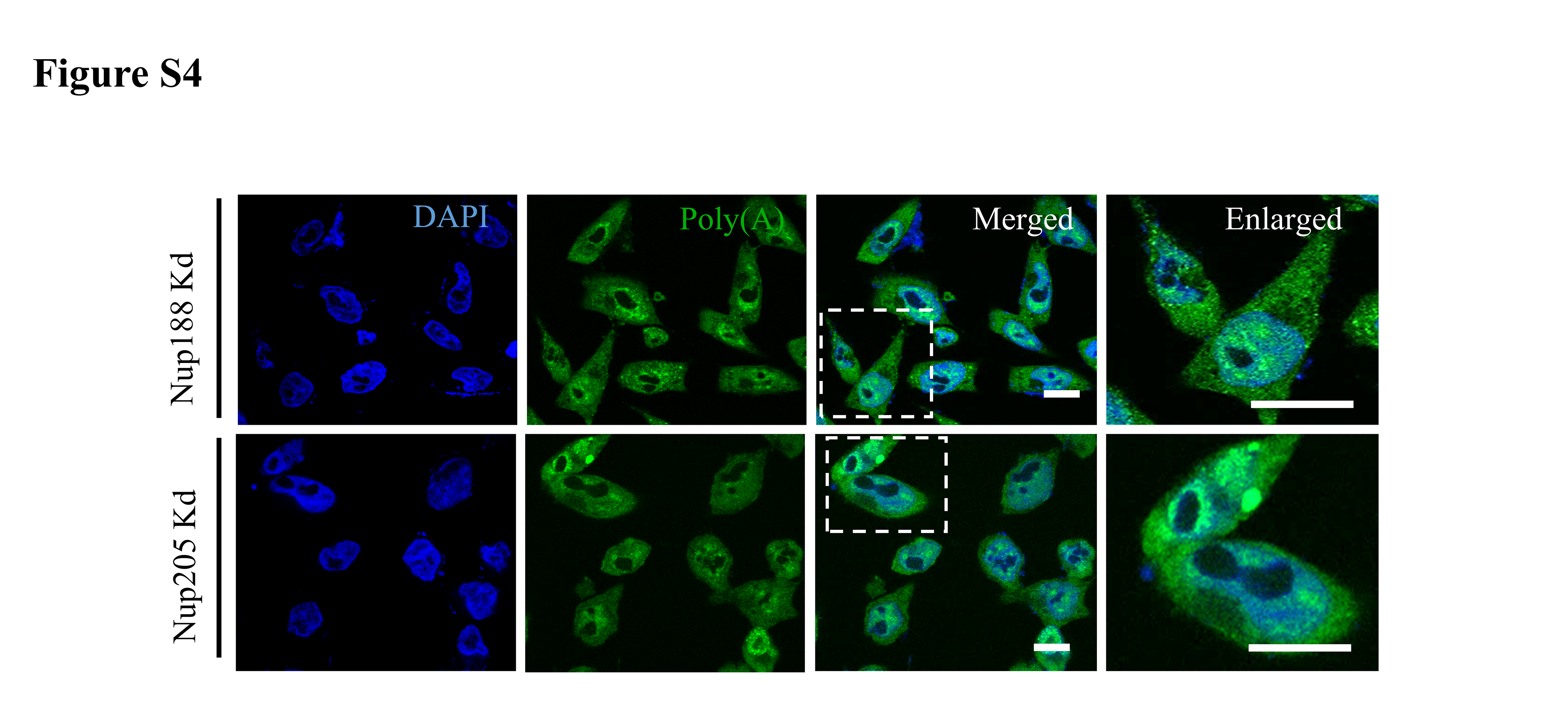

Supplement: Supplementary file 4 — Additional file 4: Figure S4. Representative images of Poly(A) RNA FISH performed using FAM labeled oligo(dT) probe (green) in Nup188 Kd and Nup205 Kd. Scale bar: ~10 µm. [file 13072_2016_106_MOESM4_ESM.tif]
