# Supplementary material for: HOXA repression is mediated by nucleoporin Nup93 assisted by its interactors Nup188 and Nup205
Source: Epigenetics Chromatin. 2016 Dec 3;9:54. doi: 10.1186/s13072-016-0106-0 (PMC5135769; doi:10.1186/s13072-016-0106-0)
Supplement: Supplementary file 5 — Additional file 5: Table S1. List of ChIP-qPCR primers. Table S2. List of RT-PCR primers. Table S3. Antibodies and their dilutions used in this study. [file 13072_2016_106_MOESM5_ESM.docx]

**Table S1:** List of ChIP-qPCR primers

| **Gene name** | **Primer pair** | **Sequence** |
| --- | --- | --- |
| HOXA1 | P1 | F-CCCGGTGCAAAACTGAGT  R-AATGGAGGGGGAAGAGCG |
|  | P2 | F-CGCTCTTCCCCCTCCATT  R-ACCGTTCAATGAAAGATGAACTG |
|  | P3 | F-CTCGCCAGTTCATCTTTCATT  R-CCTCCTGCAAAAGTTTGCC |
|  | P4 | F-AAATGCCACTAAAACGGTGATC  R-TCTTGCATTGTCCATCTGTCA |
| HOXA3 | P1 | F-TGCAGTGGTACGATCTCAGC  R-AAAATTAGCCAGGCATGGTG |
|  | P2 | F-GGTCTCGAACTGCTGACCTC  R-AGGGACCTGGAGGTATTGCT |
|  | P3 | F-TATGAGGCAGGCAGCAGTAA  R-TCTCAGGTTTGGGGGATAGA |
|  | P4 | F-TTTGGACAACCCATGAACAA  R-TGGCTATGCCTGAGTGTGAG |
| HOXA5 | P1 | F-AAGACCCCAGTAACCCGC  R-TTTGTGTAGTGTTTCTCCAAGGC |
|  | P2 | F-TGTATGGAATTTGACCTCGC  R-CAACAACTTTATTTCCCCCG |
|  | P3 | F-CGGGGGAAATAAAGTTGTTG  R-TGCACTAATAGGGGAGTTGGG |
|  | P4 | F-CCCAACTCCCCTATTAGTGCA  R-GATATGTGTGCTTGATTTGTGGC |
| GLCCI1 | P1 | F-AGCTTGTCAGTGTGGGCAG  R-TCTTGGGAGGAATAAATACCAGA |
|  | P2 | F-AAGCTATCTGCTTCGGAAAAGC  R-AATTTGCAAGTACACCTGCATCC |
|  | P3 | F-TAAAGTTTGCCTCATGTGTCCTG  R-CTCGAGTGATTGGTTCTGGAA |
| GAPDH |  | Diagenode, High Cell ChIP Kit GAPDH primer,  Cat. Kch-913-050, Lot no.- D002 |
| GRM8 |  | F- CCAGTACTTGGGGAGGTTGA  R- GCAAACCACACCTGGCTAAT |
| Outside promoter regions | Upstream region | F-ctgaaagaggcgttttgagc  R-ggagctggtctctttcaacg |
|  | Downstream region | \| F-atgaatgcagtgatgggtca \| \| --- \| \| R-aaccaagaggggagaggaaa \| |

**Table S2:** List of RT-PCR primers

| **No** | **Gene name** | **Sequence** |
| --- | --- | --- |
| 1 | Nup93 | F-AGAAGACGCCCTTGACTTTAC  R-GATATAAATTTGCCGCGCATAGG |
| 2 | Nup188 | F-CTGGGCAATCAGCAGGATATAA  R-AATGATCCCAAGGCCAGAAG |
| 3 | Nup205 | F-GACCCTAGAACTCAGTCCAGA  R-CTGTGACACCAGCGTAAGAA |
| 5 | CTCF | F-CGTTACTGTGATGCTGTGTTTC  R-TCATGTGCCTCTCCTGTCTA |
| 6 | GLCCI1 | F-GCGAACCTCCTCTTTGGATAC  R-GCTAGGTGTCTGAGTAGCTTTG |
| 7 | CFTR | F-GGGCTAATCTGGGAGTTGTTAC  R-CCAGCTCTCTGATCTCTGTACT |
| 8 | HOXA1 | F-CGTTAAATCAGGAAGCAGACCC  R-GTAGCCGTACTCTCCAACTTTC |
| 9 | HOXA2 | F-CTCAGCCACAAAGAATCCCT  R-AGCTCTAGAAGCTGTGTGTTG |
| 10 | HOXA3 | F-CTCCAGCTCAGGCGAAAG  R-CACAGGTAGCGGTTGAAGT |
| 11 | HOXA4 | F-GTCAGCGCCGTTAACCC  R-GGGTCAGGTATCGATTGAAGTG |
| 12 | HOXA5 | F-CTGCACATAAGTCATGACAACATAG  R-GGTCAGGTAACGGTTGAAGT |
| 13 | HOXA6 | F-TCCCGGACAAGACGTACAC  R-CGCCACTGAGGTCCTTATCA |
| 14 | HOXA7 | F-AGCTTGGAAATTCTGCTCACTTCT  R-TCTGATGTCATGGCCAAATTTG |
| 15 | HOXA9 | F-AAAAGCGGTGCCCCTATACA  R-CGGTCCCTGGTGAGGTACAT |
| 16 | HOXA10 | F-GAGAGCAGCAAAGCCTCGC  R-CCAGTGTCTGGTGCTTCGTG |
| 17 | HOXA11 | F-TTGAGCATGCGGGACAGTT  R-GTACCAGATCCGAGAGCTGGAA |
| 18 | HOXA13 | F-AGCGCGTGCCTTATACCAAG  R-GCCGCTCAGAGAGATTCGT |
| 19 | Nup98 | F-GCTGTTGGTTCGACCCTGTT  F-AACAGGGTCGAACCAACAGC |

**Table S3:** Antibodies and their dilutions used in this study.

| No | Antibody | Cat. Number | Western blot | IP | ChIP |
| --- | --- | --- | --- | --- | --- |
| 1 | Rabbit anti–Nup93 | sc-292099 | 1:500 | 2µg / 500 µg of protein | 2µg / 100 µg of chromatin |
| 2 | Rabbit anti-Nup188 | ab86601 | 1:1000 | 2µg / 500 µg of protein | ▬ (Not used) |
| 3 | Rabbit anti-Nup205 | HPA024574 | 1: 500 | ▬ | ▬ |
| 4 | Rouse anti–Nup98 | sc-74553 | 1:500 | 2µg / 500 µg of protein | ▬ |
| 5 | Rabbit anti-EED | ab4469 | 1:500 | ▬ | ▬ |
| 6 | rabbit anti EZH2 | ab3748 | 1:500 | ▬ | ▬ |
| 7 | rabbit anti-Suz12 | ab12073 | 1:500 | ▬ | ▬ |
| 8 | rabbit anti-CTCF | 07-729 | 1:500 | ▬ | ▬ |
| 9 | Rabbit anti-H3K9Ac | ab4441 | 1:5000 | ▬ | 2µg / 100 µg of chromatin |
| 10 | Mouse anti 3K27me3 | ab6002 | 1:1000 | ▬ | 2µg / 100 µg of chromatin |
| 11 | Anti-pan H3 | ab1791 | 1:5000 | ▬ | 2µg / 100 µg of chromatin |
| 12 | Anti-H3K36me3 | ab9050 | 1:1000 | ▬ | 2µg / 100 µg of chromatin |
| 13 | Anti-GAPDH | G9545 | 1:5000 | ▬ | ▬ |
